# Efferocytosis related KCTD12 is a clinico-immune target in lung adenocarcinoma

**DOI:** 10.3389/fonc.2026.1930176

**Published:** 2026-08-19

**Authors:** Wen Shan, Guanya Cao, Yu Liu, Yifei Zhu

**Affiliations:** 1 Department of Thoracic Surgery and Cardiac Surgery, The Second People’s Hospital of Fuyang City, Fuyang, China; 2 Department of Pancreatic Surgery, Fudan University Shanghai Cancer Center, Shanghai, China; 3 Department of Oncology, Shanghai Medical College, Fudan University, Shanghai, China

**Keywords:** efferocytosis, immunotherapy, KCTD12, lung adenocarcinoma, tumor microenvironment

## Abstract

**Background:**

Efferocytosis, the clearance of apoptotic cells by phagocytes, contributes to immune homeostasis but may also promote tumor immune tolerance. However, its transcriptional landscape and clinical relevance in lung adenocarcinoma (LUAD) remain incompletely understood.

**Methods:**

We systematically analyzed efferocytosis-associated genes across TCGA and multiple GEO datasets to classify LUAD subtypes and construct a prognostic risk model. The prognostic and immunological relevance of this model was validated in four independent cohorts and further assessed through immune infiltration, genomic, and immunotherapy datasets. Functional and pharmacogenomic analyses were performed to identify potential therapeutic vulnerabilities, and the efferocytosis-associated signature gene *KCTD12* was subsequently validated *in vitro.*

**Results:**

Unsupervised clustering identified two efferocytosis-based LUAD subtypes with distinct prognostic and immune–metabolic characteristics. The derived risk model robustly predicted overall survival across validation cohorts. Among the model genes, KCTD12 emerged as an efferocytosis-associated candidate linked to an immune-active tumor microenvironment. Across the analyzed single-cell, spatial transcriptomic, and immunotherapy-treated cohorts, higher KCTD12 expression was associated with enhanced cytotoxic T-cell activity and more favorable treatment outcomes. Functional experiments confirmed that KCTD12 suppresses tumor cell proliferation, reduces colony formation, and enhances OT-1 CD8^+^ T-cell activation and cytotoxicity.

**Conclusions:**

Our study identifies an efferocytosis-associated transcriptional program linked to immune heterogeneity and prognosis in LUAD. The efferocytosis-related risk signature provides a framework for prognostic and immune stratification, while KCTD12 represents a candidate biomarker associated with immune activation and clinical outcomes in immunotherapy-treated cohorts. Its treatment-specific predictive value requires prospective validation in appropriately controlled studies.

## Introduction

Lung adenocarcinoma (LUAD) is the most prevalent histological subtype of non–small cell lung cancer (NSCLC) and remains a leading cause of cancer-related mortality worldwide (1). Although immune checkpoint blockade (ICB) has substantially improved clinical outcomes for a subset of patients, therapeutic responses remain highly heterogeneous, highlighting the need for biomarkers that better reflect the tumor immune microenvironment and predict immunotherapy benefit (2).

Efferocytosis, the process by which phagocytes remove apoptotic cells, is essential for maintaining tissue homeostasis and immune balance (3–5). In the tumor microenvironment, dysregulated efferocytosis may contribute to immune suppression by promoting anti-inflammatory signaling and limiting effective antigen presentation (6–9). Despite increasing evidence implicating efferocytosis in tumor progression, its transcriptional characteristics and clinical significance in LUAD remain poorly defined.

In this study, we systematically characterized efferocytosis-associated transcriptional patterns in LUAD using multi-omics and transcriptomic datasets. We identified two molecular subtypes with distinct immune and metabolic features and developed an efferocytosis-associated prognostic model that was evaluated in multiple independent cohorts. Integrative analyses revealed associations between the risk score, tumor immune infiltration, genomic alterations, and outcomes in immunotherapy-treated datasets. KCTD12 was subsequently identified as a candidate signature gene associated with cytotoxic T-cell activity and clinical outcomes among patients receiving immune checkpoint blockade. Functional assays further showed that modulation of KCTD12 altered tumor-cell proliferation and susceptibility to antigen-specific CD8^+^ T-cell killing. These findings support further investigation of KCTD12 as an immune-associated candidate biomarker, while its treatment-specific predictive value remains to be established.

## Materials and methods

### Data collection

The study included LUAD patients from TCGA (n = 500) with available RNA-seq data (FPKM format converted to TPM), complete clinical annotations, and survival data. External validation cohorts (GSE31210, GSE37745, GSE50081, GSE68465) were selected based on the availability of comparable expression and survival data. Exclusion criteria included missing survival information, poor-quality sequencing data. Normal adjacent tissues were excluded from survival analysis but included for differential expression comparisons. The training cohort included the transcriptome data and clinical information of 500 patients with LUAD were retrieved from the Cancer Genome Atlas (TCGA) database (https://portal.gdc.cancer.gov/). Validation sets were obtained from GSE31210, GSE37745, GSE50081, GSE68465 expression profiles from the Gene Expression Omnibus (GEO) database (http://www.ncbi.nlm.nih.gov/geo) (10–13). The complete efferocytosis-related gene details are displayed in **Supplementary Table 1**. Single-cell RNA sequencing data were obtained from GSE207422 (14). The RNA-seq sample expression levels were converted from fragments per kilobase of transcript per million mapped reads (FPKM) to transcripts per million (TPM), and then log2(TPM + 1) was calculated. Somatic mutation data, copy number variation (CNV) files, and tumor mutation burden (TMB) data of LUAD patients were retrieved from the TCGA database.

### Construction and validation of efferocytosis-related prognostic signature

We identified 167 efferocytosis-related genes and performed univariate analysis to select those significantly associated with patient survival (p<0.05) (**Supplementary Table 2**). Subsequently, least absolute shrinkage and selection operator (LASSO) regression and multivariate Cox analysis were applied to refine gene selection and calculate risk coefficients. Using tenfold cross-validation, the optimal regularization parameter was determined. Genes with non-zero coefficients were considered potential prognostic markers, leading to the development of the Efferocytosis-Related Prognostic score model:


Efferocytosis−Related Prognostic score=∑(Expi∗coefi)

where coefi represents the regression coefficient of gene i derived from the training cohort, and Expi represents the normalized expression level of the corresponding gene. Patients in the training cohort (n = 500) were stratified into high- and low-risk groups based on the median Efferocytosis-Related Prognostic score. Kaplan-Meier survival analysis was conducted to assess survival outcomes between the groups (**Supplementary Table 3**). Pairwise correlations among the 12 genes included in the final prognostic signature were assessed using Spearman correlation analysis. Multicollinearity was further evaluated using variance inflation factors, with a VIF greater than 5 considered indicative of potentially problematic collinearity. Internal model validation was performed using 1,000 bootstrap resamples. Model optimism was estimated as the difference between the apparent performance in each bootstrap sample and the performance of the bootstrap-derived model when applied to the original cohort. The optimism-corrected Harrell C-index was subsequently calculated. The median cutoff was prespecified in the TCGA training cohort and applied to the validation cohorts without cohort-specific optimization. Cutoff robustness was additionally assessed using the 40th, 50th, and 60th percentiles of the training-cohort risk-score distribution. Raw RNA-sequencing count data were converted to transcripts per million (TPM), followed by log2(TPM + 1) transformation. Gene symbols were standardized according to the HGNC nomenclature, and duplicate gene symbols were summarized using mean expression. The same preprocessing procedure was applied to the training and external validation cohorts before risk-score calculation. In the training cohort, patients were classified into high- and low-risk groups according to the median risk score. The same fixed cutoff was subsequently applied to all external validation cohorts without recalibration or cohort-specific optimization. For each external validation cohort, the risk score was calculated using the regression coefficients derived exclusively from the training cohort. The model was not refitted, and no coefficients were re-estimated in the validation datasets. Patients were stratified using the prespecified cutoff derived from the training cohort. Kaplan–Meier survival analysis and time-dependent receiver operating characteristic analysis were then performed to evaluate the prognostic performance of the model. The model’s predictive performance was evaluated through ROC curves, while the AUC was calculated to quantify accuracy. This risk signature was further validated in three independent GEO datasets (GSE31210, GSE37745, GSE50081, GSE68465), where survival analysis and ROC analysis confirmed the robustness of the Efferocytosis-Related Prognostic score model.

### Development and validation of a nomogram scoring system

To determine the independent prognostic values and the predictive efficacy of the Efferocytosis-Related Prognostic score in predicting the survival of LUAD patients, we conducted univariable and multivariate Cox regression analyses for better clinical practice. Furthermore, we investigated the relationship between the Efferocytosis-Related Prognostic score and various clinical characteristics. Additionally, in order to improve the prognostic accuracy of our model, a nomogram was developed utilizing the risk score, M stage, and age as independent prognostic factors to estimate the probability of overall survival (OS) at 1-, 3-, and 5-years.

### Relationship between risk groupings and clinical characteristics

The study analyzed clinical factors such as age, gender, and TNM stage. Differences in prognostic outcomes were assessed using Kaplan-Meier analysis in R software with the “survival” and “survminer” packages (15).

### Functional and pathway enrichment analysis

To identify specific biological pathways enriched between the high- and low-risk groups, we performed a comprehensive analysis using various techniques. Initially, we conducted Gene Ontology (GO) analysis and Kyoto Encyclopedia of Genes and Genomes (KEGG) analysis to understand the functions of the screened candidate genes and related pathways (16, 17).

Gene Set Enrichment Analysis (GSEA) was utilized to identify enriched pathways or gene sets based on differential expression results between the high and low groups. Following this, we utilized the “clusterProfiler” packages for further elucidation (18).

### Exploration of the immune landscape in distinct risk groupings

The immune characteristics of 500 LUAD samples were assessed by evaluating the scores of TME cells using the single sample gene set enrichment analysis (ssGSEA) algorithm. To analyze the relative proportion of 22 immune cells within the LUAD samples, CIBERSORT (https://cibersort.stanford.edu) was employed. By leveraging the 500 samples gene expression matrix and the provided gene expression feature set of the 22 immune cell subtypes from the official website, simulation calculations were iterated 1000 times to derive the relative composition ratio of the 22 immune cells in each sample. Subsequently, the immune score and ESTIMATE score of each patient were assessed utilizing the R package of estimate (19).

### Mutation analysis

To characterize the somatic mutation landscape between the high- and low-risk LUAD groups, mutation annotation format (MAF) files from TCGA were analyzed using the “maftools” R package. Tumor mutation burden (TMB) was calculated for each patient and compared between the two risk groups (20).

### Patient-level single-cell analysis

Single-cell RNA-seq data from the NSCLC immunotherapy cohort GSE207422 were processed using the “Seurat” R package. Cells expressing 200–5,000 genes and containing less than 10% mitochondrial transcripts were retained for downstream analysis (21). Major cell populations were annotated according to the original dataset annotations and evaluated using canonical lineage markers. Malignant cells were identified according to the original tumor-cell annotation and supported by epithelial and tumor-associated marker expression. To quantify patient-level KCTD12 expression, raw transcript counts from malignant cells belonging to the same patient were aggregated to generate pseudobulk expression profiles. Patients with fewer than 20 malignant cells were excluded to reduce instability caused by sparse malignant-cell sampling. Pseudobulk counts were normalized using the trimmed mean of M-values method and transformed to logCPM values. The remaining 15 patients were stratified at the median patient-level KCTD12 expression into KCTD12-low (n = 7) and KCTD12-high (n = 8) groups.

Major cell-type proportions were calculated independently for each patient. When multiple samples were available from the same patient, proportions were first calculated within each sample and then averaged to obtain one value per patient. CD8^+^ T-cell-subtype proportions were calculated analogously within the CD8^+^ T-cell compartment. Group comparisons were performed using two-sided Wilcoxon rank-sum tests, and correlations were evaluated using Spearman correlation analysis. Benjamini–Hochberg correction was applied separately across the major cell-type and CD8^+^ T-cell-subtype analyses. Individual cells were not treated as independent statistical observations.

Cell–cell communication analysis was performed using “CellChat” to infer ligand–receptor interactions, incoming and outgoing signaling patterns, and communication strength between malignant cells and immune or stromal populations. CD8^+^ T-cell subsets were further annotated to evaluate differences in naïve, cytotoxic, proliferative, stressed, exhausted, and GZMK^+^ CD8^+^ T-cell populations according to KCTD12 expression.

### Spatial transcriptomics data analysis

We downloaded two datasets of Visium spatial transcriptomics (ST) from hepatocellular carcinoma patients treated with anti-PD1 (22, 23). To compare the expression of KCTD12 between responders and non-responders, we aggregated the counts of all spatial spots to generate a pseudo-bulk for each ST sample, normalizing the data to log2(TPM + 1). The expression level of *KCTD12* in ST samples was visualized using the R package SpaCET (24).

### Quantitative real-time PCR assay

Total RNA was extracted from LLC cells using TRIzol reagent following the manufacturer’s protocol. RNA was reverse transcribed to cDNA using ABScript II RT Mix (ABclonal, Wuhan, China). qPCR was performed using TB Green Premix Ex Taq II (TaKaRa, Japan) on ROCHE LightCycler^®^480 system. The relative expression of target genes was normalized to ACTIN and calculated using the 2^−ΔΔCt method. All reactions were conducted in triplicate.

### Mice

OT-1 TCR transgenic mice were (female, 6–8 weeks old) were purchased from National Rodent Laboratory Animal Resources (Shanghai, China). All mice were maintained under specific pathogen-free conditions at 20–22 °C, ~60% humidity, and a 12-h light/dark cycle. All protocols for mouse experiments were approved by the Ethics Committee at Fudan University Shanghai Cancer Center.

### Cell proliferation assay

Cell proliferation was assessed using the CCK8 assay. Briefly, transfected LLC cells were seeded into 96-well plates at a density of 2 × 10^3 cells/well and incubated at 37 °C with 5% CO2. At indicated time points, 10 μL of CCK8 reagent was added to each well and incubated for 2 hours. Absorbance was measured at 450 nm using a microplate reader.

### Colony formation assay

For colony formation assay, 4000 cells per well were plated in a 6-well plate in triplicate and cultured for 14 days before staining viable colonies with crystal violet.

### Immunoblotting

For protein analysis, cell pellets were harvested and lysed in RIPA lysis buffer containing phosphatase and protease inhibitor cocktail (MedChemExpress). Protein concentration was quantified by the BCA Protein Assay Kit (Thermo Fisher Scientific). According to the quantified results, the loading amount of each sample was adjusted to be consistent. Samples were electrophoresed with 8–15% SDS–PAGE gel and transferred to a nitrocellulose membrane. The membrane was blocked with 5% BSA for 1 h, and incubated with indicated primary antibodies at 4 °C overnight. After probing with corresponding secondary antibodies, proteins were visualized by LI-COR Odyssey Infrared Imaging System (LI-COR Biosciences).

### *In vitro* OVA-specific cytotoxic CD8 + T cell killing assay

Splenocytes derived from OT-1 TCR transgenic mice were cultured in complete RPMI 1640 medium (containing 10% fetal bovine serum, antibiotics, 2-mercaptoethanol (0.05 mM) and IL-2 (10 ng ml−1)) plus OVA peptide (10 nM). 48 h later, cells were washed to remove OVA and cultured in complete RPMI 1640 medium for another 72 h. OT1 CTLs were then resuspended in 1640 medium and cocultured with cancer cells stably expressing OVA for 12 h.

### *In vitro* cytotoxicity assay

Tumor cells stably expressing CD19 were plated onto 96-well plates. CD19 CAR-T cells were then co-cultured with tumor cells for 24 h in the complete T cell medium. Tumor cells post co-culture were analyzed by CellCounting-Lite 2.0 Luminescent Cell Viability Assay (Vazyme) for cell viability. Data were collected and analyzed by EnSight.

### T cell co-culture screen and assay

1×10^5^ LLC-OVA tumor cells were seeded per well in the 96-well plate with DMEM complete medium and pre-incubated for 12 h. OT-1 CD8+T cells were isolated from the spleens of OT-1 mice using EasySep Mouse CD8+T Cell Isolation Kit. The purified OT-1 CD8+T cells were co-cultured with tumor cells at a ratio of 5:1 for 12 h in complete medium containing IL-2–4 h before cell collection, Brefeldin A (1:1000) was added to block cytokine secretion. T cells were washed and resuspended in staining buffer and stained with anti-CD8b-Percp/Cyanine5.5 for 30 min on ice. After washing, cells were performed intracellular staining with anti-IFN-γ-APC, anti-TNF α-BV421 and anti-Granzyme B-PE.

### Statistical analysis

Statistical analyses were performed using appropriate tests based on data distribution. For comparisons between two groups, unpaired Student’s t-tests were applied to normally distributed data, while Mann–Whitney U tests were used for non-normally distributed data. One-way ANOVA and Kruskal–Wallis tests assessed differences across multiple groups. Correlations between variables were analyzed using Pearson’s or Spearman’s correlation tests. Survival differences between high- and low-risk groups were evaluated via Kaplan–Meier analysis, with significance tested by the Log-rank test. ROC curves were generated to assess the model’s performance, and AUC values were calculated. All statistical analyses were carried out using R software (version 4.3.2) and GraphPad Prism 9, with significance set at p < 0.05.

## Results

### Identification of efferocytosis-based molecular subtypes in LUAD

**Figure 1:**
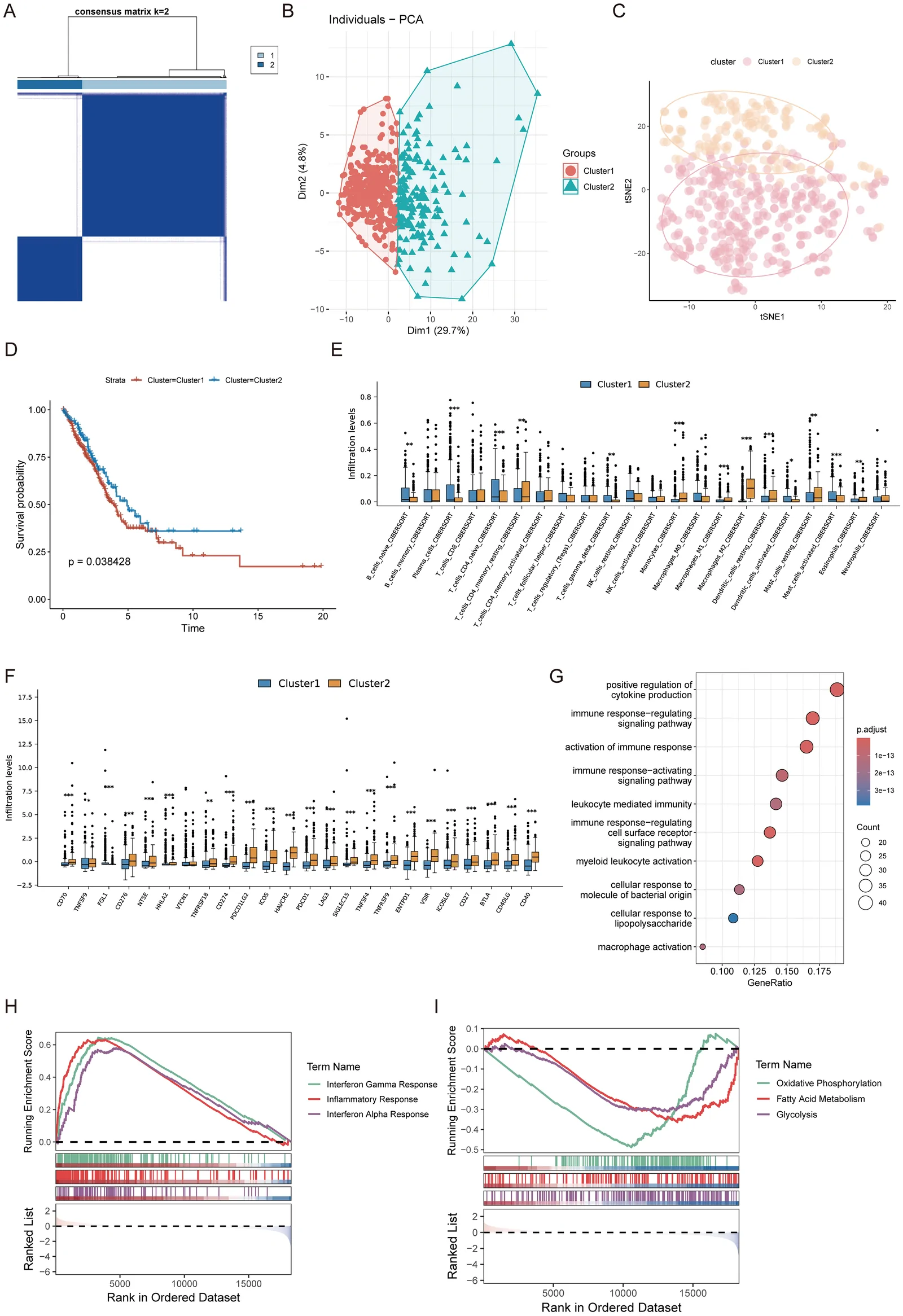
Efferocytosis-based clustering reveals two LUAD subtypes with distinct prognosis and immune characteristics. **(A)** Consensus matrix heatmap showing optimal clustering stability at *k* = 2 based on efferocytosis-related gene expression in TCGA-LUAD. **(B)** Principal component analysis (PCA) plot displaying separation between Cluster 1 and Cluster 2. **(C)** t-distributed stochastic neighbor embedding (t-SNE) visualization of the two clusters. **(D)** Kaplan–Meier curves for overall survival (OS) between Cluster 1 and Cluster 2 (log-rank *p* = 0.038). **(E, F)** Comparison of immune cell infiltration levels across clusters estimated by ssGSEA. **(G)** GO enrichment analysis of genes upregulated in Cluster 2 showing immune activation–related terms; bubble size represents gene count, and color indicates adjusted *p* value. **(H)** GSEA plots demonstrating enrichment of Interferon-γ response, Interferon-α response, and inflammatory response pathways in Cluster 2. **(I)** GSEA plots showing enrichment of oxidative phosphorylation, fatty acid metabolism, and glycolysis in Cluster 1. * indicates p < 0.05, ** indicates p < 0.01, and *** indicates p < 0.001.

To investigate the biological heterogeneity of LUAD driven by efferocytosis-related genes, we conducted consensus clustering using the TCGA-LUAD cohort. The cumulative distribution function (CDF) and consensus matrix indicated optimal clustering stability at *k* = 2, thereby defining two distinct molecular subtypes (Cluster 1 and Cluster 2) (**Figure 1A**). Principal component analysis (PCA) and t-distributed stochastic neighbor embedding (t-SNE) further confirmed a clear separation between the two clusters (**Figures 1B, C**). Kaplan–Meier survival analysis revealed that Cluster 1 exhibited significantly poorer overall survival compared with Cluster 2 (log-rank *p* = 0.038; **Figure 1D**), underscoring the prognostic significance of efferocytosis-associated transcriptional programs in LUAD.

We next characterized the tumor immune microenvironment across clusters. Cluster 1 displayed globally higher immune and stromal infiltration scores across multiple deconvolution algorithms, accompanied by increased abundance of CD8^+^ T cells, NK cells, macrophages, and dendritic cells (**Figures 1E, F**). Functional enrichment of genes upregulated in Cluster 2 revealed immune-activation signatures, including the positive regulation of cytokine production, immune response–regulating signaling pathways, and macrophage activation (**Figure 1G**). Gene Set Enrichment Analysis (GSEA) further demonstrated that Cluster 2 was significantly enriched in interferon-γ response, interferon-α response, and inflammatory response pathways (**Figure 1H**), whereas Cluster 1 exhibited pronounced activation of metabolic programs, including oxidative phosphorylation, fatty acid metabolism, and glycolysis (**Figure 1I**).

### Construction and validation of an efferocytosis-related prognostic signature in LUAD

**Figure 2:**
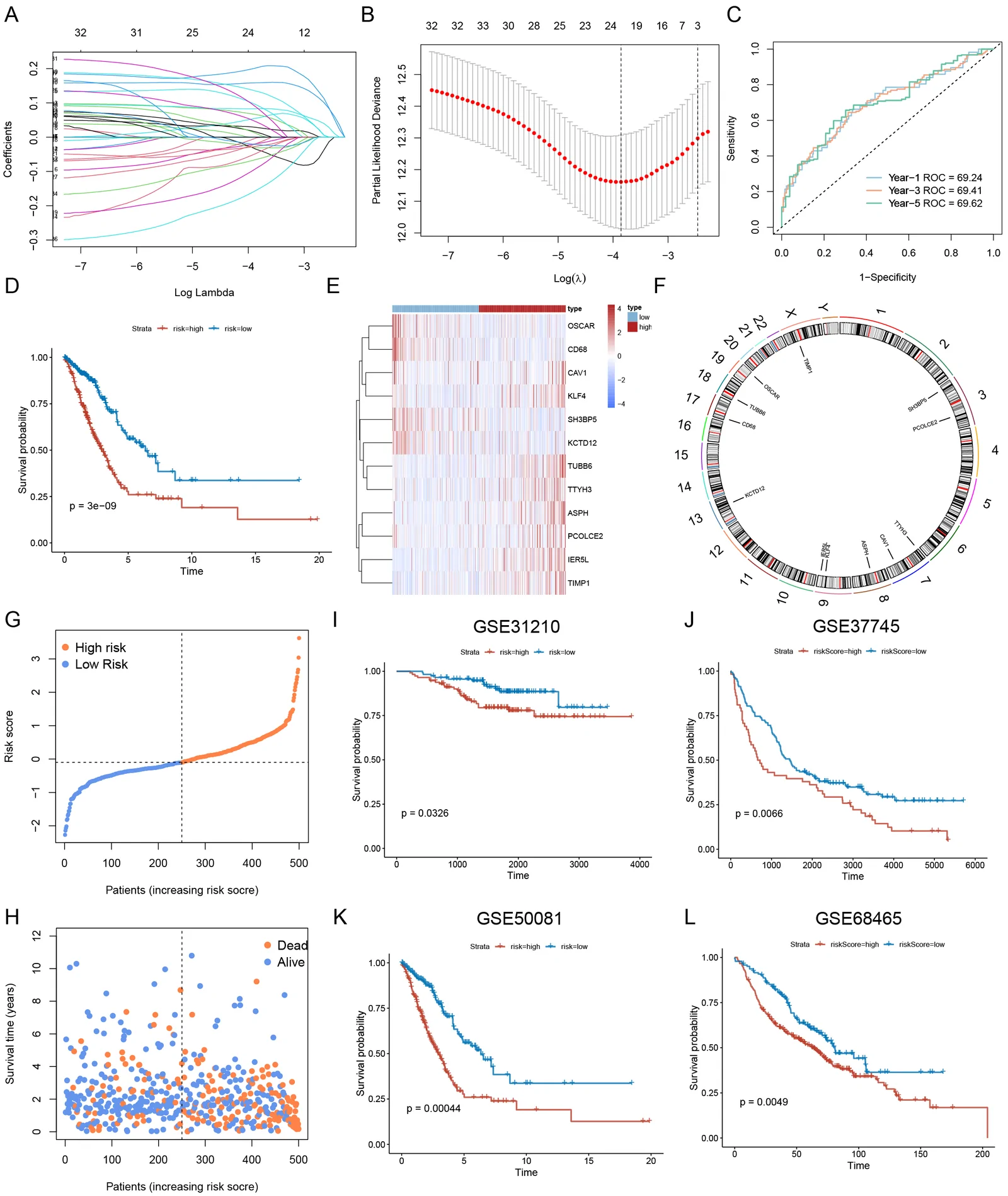
Construction and validation of the efferocytosis-related prognostic model in LUAD. **(A–B)** LASSO Cox regression analysis identifying optimal penalty parameter (λ) and selected variables. **(C)** Time-dependent ROC curves at 1-, 3-, and 5-year overall survival (OS) showing predictive accuracy of the model. **(D)** Kaplan–Meier survival curves of high- and low-risk LUAD patients in the TCGA cohort (log-rank *p* < 3 × 10^-9^). **(E)** Heatmap of 12 efferocytosis-related prognostic genes across risk groups. **(F)** Circos plot showing chromosomal localization of model genes. **(G, H)** Distribution of risk scores and survival status of patients in TCGA-LUAD. **(I–L)** Validation of the prognostic model in four external GEO datasets (GSE31210, GSE37745, GSE50081, and GSE68465). Two-sided log-rank tests were used; *p* < 0.05 was considered significant.

To further quantify the prognostic potential of efferocytosis-associated genes, we conducted univariate Cox regression and least absolute shrinkage and selection operator (LASSO) analyses in the TCGA-LUAD cohort. Twelve genes (*OSCAR, CD68, CAV1, KLF4, SH3BP5, KCTD12, TUBB6, TTYH3, ASPH, PCOLCE2, IER5L*, and *TIMP1*) were ultimately retained to construct a multigene prognostic signature (**Figures 2A, B**; **Supplementary Figure S1**). Patients were stratified into high- and low-risk groups according to the median risk score. Kaplan–Meier survival analysis revealed that the high-risk group exhibited significantly shorter overall survival than the low-risk group (log-rank *p* < 3 × 10^-9^; **Figure 2D**). Time-dependent receiver operating characteristic (ROC) analysis demonstrated favorable predictive performance of the model at 1-, 3-, and 5-year intervals (AUC = 0.6924, 0.6941, and 0.6962, respectively; **Figure 2C**).

The heatmap illustrated distinct expression profiles of the signature genes between risk groups (**Figure 2E**), while their chromosomal distribution was visualized in a circos plot (**Figure 2F**). The distribution of risk scores and corresponding survival status revealed a clear trend of increasing mortality with elevated risk scores (**Figures 2G, H**). Notably, the prognostic value of the efferocytosis-related gene signature was validated across four independent GEO datasets (GSE31210, GSE37745, GSE50081, and GSE68465), each consistently demonstrating poorer survival outcomes among high-risk patients (log-rank *p* = 0.0326, 0.0066, 0.00044, and 0.0049, respectively; **Figures 2I–L**). In TCGA-LUAD, the maximum absolute pairwise correlation among the 12 signature genes was 0.498 (OSCAR–CD68), and the maximum VIF was 1.689. The apparent C-index of the refitted 12-gene Cox model was 0.670; after 1,000 bootstrap resamples, mean optimism was 0.019 and the optimism-corrected C-index was 0.652 (bootstrap-derived 95% interval 0.606–0.696). High- versus low-risk HRs at the 40th, 50th, and 60th percentile cutoffs were 2.21 (95% CI 1.58–3.09), 2.50 (95% CI 1.83–3.41), and 2.50 (95% CI 1.86–3.37), respectively (**Supplementary Figure 2**; **Supplementary Table 4**). External performance was heterogeneous. In GSE31210, the high-risk group had poorer overall survival (HR = 2.11, 95% CI 1.05–4.24), with a C-index of 0.628 (95% CI 0.517–0.722) and 1-, 3-, and 5-year AUCs of 0.784, 0.631, and 0.608. In the adenocarcinoma subsets of GSE37745 and GSE50081, the HRs were 1.60 (95% CI 1.14–2.25) and 2.25 (95% CI 1.43–3.54), with C-indexes of 0.525 and 0.541, respectively. In the GSE68465 sensitivity analysis, the median-dichotomized HR was 1.52 (95% CI 1.13–2.03), the C-index was 0.570.

Collectively, these results establish the efferocytosis-related gene signature as a robust and reproducible tool for prognostic stratification in LUAD.

### Clinical validation and independent prognostic value of the efferocytosis-related risk model in LUAD

**Figure 3:**
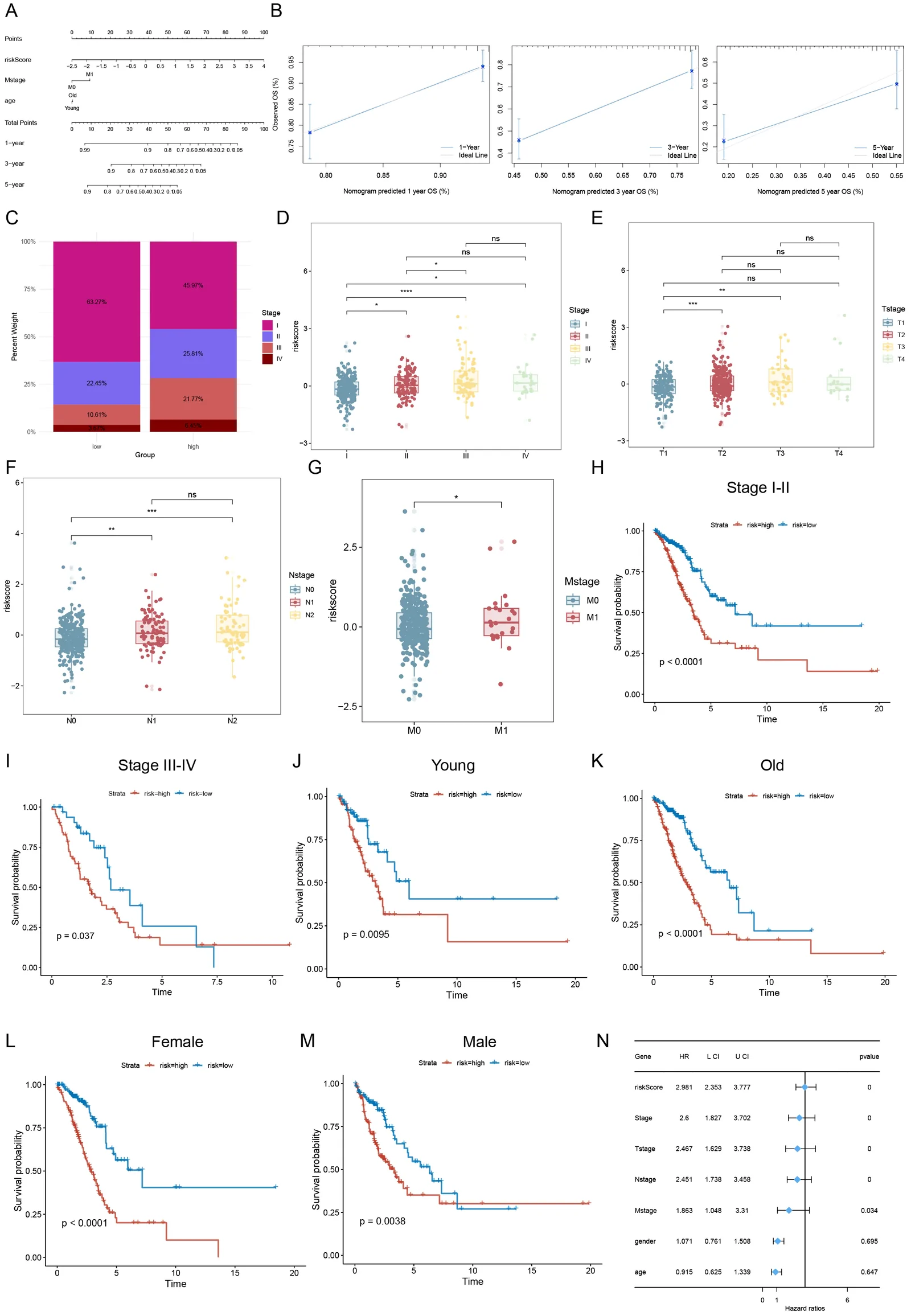
Clinical validation and prognostic independence of the efferocytosis-related risk model in LUAD. **(A)** Nomogram combining risk score and clinicopathologic variables (age, gender, and TNM stage) for predicting 1-, 3-, and 5-year OS. **(B)** Calibration plots confirming the nomogram’s predictive accuracy at 1, 3, and 5 years. **(C)** Proportion of TNM stages in high- and low-risk groups. **(D–G)** Distribution of risk scores across T, N, and M stages; comparisons performed using Wilcoxon tests. **(H–M)** Kaplan–Meier survival analyses stratified by clinical subgroups, including disease stage (I–II, III–IV), age (young, old), and sex (female, male). **(N)** Univariate Cox regression analysis identifying the risk score as a significant predictor of overall survival. p values: < 0.05, < 0.01, *< 0.001, **< 0.0001; ns, not significant. *p < 0.05; **p < 0.01; ***p < 0.001; ****p < 0.0001; ns, not significant.

To assess the clinical applicability of the efferocytosis-related risk model, we constructed a nomogram integrating the risk score with key clinicopathologic variables, including age and M stage (**Figure 3A**). Within the nomogram, each variable was assigned a point value, and the total score was mapped to the corresponding survival probability. The relative point range assigned to the risk score indicated that it contributed substantially to the prognostic estimation. Calibration analyses showed that the predicted 1-, 3-, and 5-year survival probabilities were generally close to the observed outcomes, with the calibration curves remaining near the 45° reference line (**Figure 3B**). These results suggest acceptable agreement between the nomogram-derived predictions and actual survival outcomes in the analyzed cohort.

Analysis of clinicopathologic distributions revealed that patients in the high-risk group were more frequently classified into advanced TNM stages (III–IV) compared with those in the low-risk group (**Figure 3C**). Consistently, the risk score increased significantly with advancing T, N, and M stages (**Figures 3D–G**), indicating that the efferocytosis-derived signature is closely associated with tumor progression. Survival stratification analyses further confirmed that the high-risk group exhibited consistently poorer OS across multiple clinical subgroups, including early-stage (I–II; *p* < 0.0001) and advanced-stage (III–IV; *p* = 0.037) disease, younger (<65 years; *p* = 0.0095) and older (≥65 years; *p* < 0.0001) patients, as well as both female and male cohorts (*p* < 0.0001 and *p* = 0.0038, respectively; **Figures 3H–M**).

Univariate Cox regression analysis revealed that the risk score, T stage, N stage, M stage, and overall clinical stage were all significantly associated with OS (**Figure 3N**). Subsequent multivariate Cox analysis (**Supplementary Figure 4A**) identified the risk score as an independent prognostic factor (HR = 2.591, 95% CI: 2.028–3.312, *p* < 0.001), even after adjustment for traditional clinical parameters. Furthermore, the high-risk group exhibited a significantly higher tumor mutation burden (TMB) compared with the low-risk group (Wilcoxon *p* = 0.0037; **Supplementary Figure 4B**), suggesting that a higher efferocytosis-related risk score may be associated with increased genomic instability in LUAD.

Collectively, these findings demonstrate that the efferocytosis-based risk model not only reflects disease aggressiveness but also serves as an independent and clinically meaningful predictor of patient outcomes in LUAD.

### Functional characterization of the risk groups highlights immune-competent biology in low risk and proliferative–invasive programs in high risk LUAD

**Figure 4:**
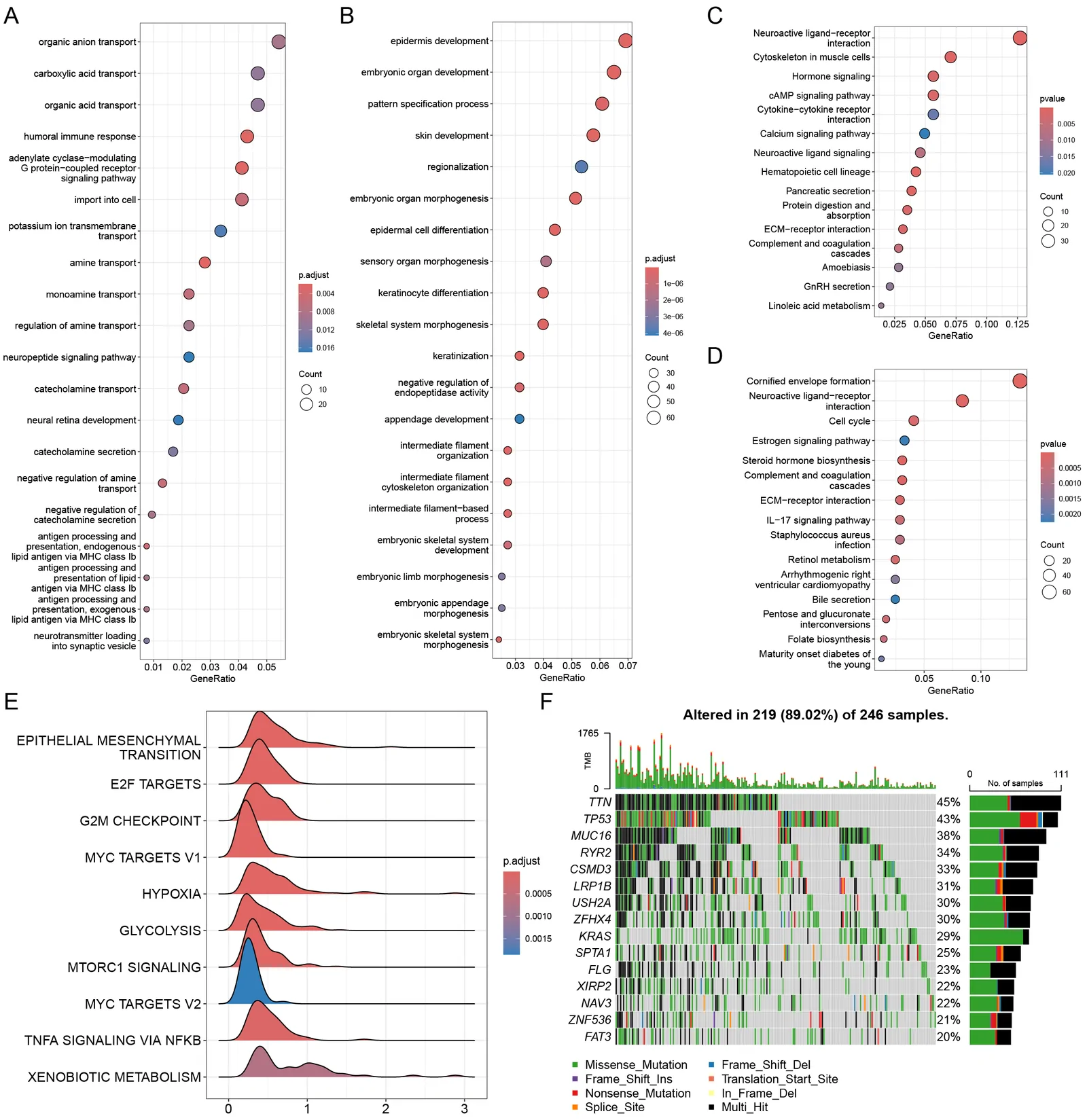
Functional and genomic differences between low- and high-risk groups. **(A)** GO enrichment for genes upregulated in the low-risk group, highlighting immune communication and antigen presentation (e.g., *humoral immune response*, *antigen processing and presentation*, GPCR/amine transport). **(B)** GO enrichment for genes upregulated in the high-risk group, emphasizing development/morphogenesis programs linked to invasiveness and tissue remodeling. **(C)** KEGG pathways enriched in low-risk tumors (e.g., *cytokine–cytokine receptor interaction*, *neuroactive ligand–receptor interaction*, *calcium/cAMP signaling*), consistent with immune/physiologic signaling. **(D)** KEGG pathways enriched in high-risk tumors (e.g., *cell cycle*, *ECM–receptor interaction*, *focal adhesion*, *IL-17 signaling*, *complement and coagulation cascades*), indicative of proliferation and pro-metastatic microenvironment. **(E)** GSEA of hallmark gene sets in the high-risk group showing enrichment of *E2F targets*, *G2M checkpoint*, *MYC targets*, *EMT*, *mTORC1 signaling*, *TNFα signaling via NF-κB*, *hypoxia*, and *glycolysis*. Curves are ordered by normalized enrichment score; color encodes adjusted *p* value. **(F)** Oncoplot waterfall for the high-risk group (TCGA-LUAD) displaying somatic mutation landscape; bar plots denote per-sample mutation counts and per-gene alteration frequency. Bubble size represents gene count; color depicts adjusted *p* value (Benjamini–Hochberg). Two-sided tests; FDR < 0.05 was considered significant.

To elucidate the biological basis underlying the survival stratification by the efferocytosis-derived score, we compared functional landscapes between the two risk groups. The low-risk subgroup was enriched for immune communication and antigen presentation processes, including antigen processing and presentation, humoral immune response, and cytokine–cytokine receptor interaction (**Figures 4A, C**). The enrichment of MHC-mediated antigen processing implies preserved tumor immunogenicity and efficient T-cell priming, typically associated with effective immune surveillance and favorable clinical outcomes. Additional overrepresentation of neuropeptide/amine transport and G-protein–coupled receptor signaling pathways suggests a more differentiated epithelial phenotype with maintained homeostatic signaling rather than oncogenic reprogramming.

In contrast, the high-risk subgroup exhibited coordinated activation of proliferative, invasive, and inflammation-driven programs across GO, KEGG, and GSEA analyses (**Figures 4B, D, E**). Enriched GO/KEGG terms—such as cell cycle, ECM–receptor interaction, focal adhesion, and IL-17 signaling—reflect accelerated cell-cycle progression and microenvironmental remodeling that promote migration and metastasis. GSEA further revealed hallmark pathways including E2F targets, G2M checkpoint, MYC targets, EMT, mTORC1 signaling, TNFα–NF-κB, hypoxia, and glycolysis, collectively defining a proliferative and hypoxia-adapted metabolic phenotype coupled with chronic inflammatory signaling. This axis is classically associated with immune exclusion, anabolic growth, and therapeutic resistance, offering a mechanistic explanation for the inferior prognosis observed in the high-risk group.

Consistent with these signatures of biological aggressiveness, high-risk tumors harbored frequent genomic alterations (**Figure 4F**), including recurrent mutations in *TP53, KRAS, LRP1B, ZFHX4, CSMD3*, and *MUC16*, among others. *TP53* and *KRAS* lesions are well-established drivers of genomic instability and malignant progression in LUAD, corroborating the adverse outcomes observed in this cohort. Collectively, the functional and genomic features converge on a model wherein the low-risk state reflects an immune-competent, antigen-presenting phenotype, whereas the high-risk state embodies a proliferative, EMT-prone, metabolically rewired phenotype characterized by heightened inflammatory signaling and mutational burden—providing a coherent mechanistic rationale for the divergent prognoses.

### The efferocytosis-related signature stratifies distinct immune infiltration patterns in LUAD

**Figure 5:**
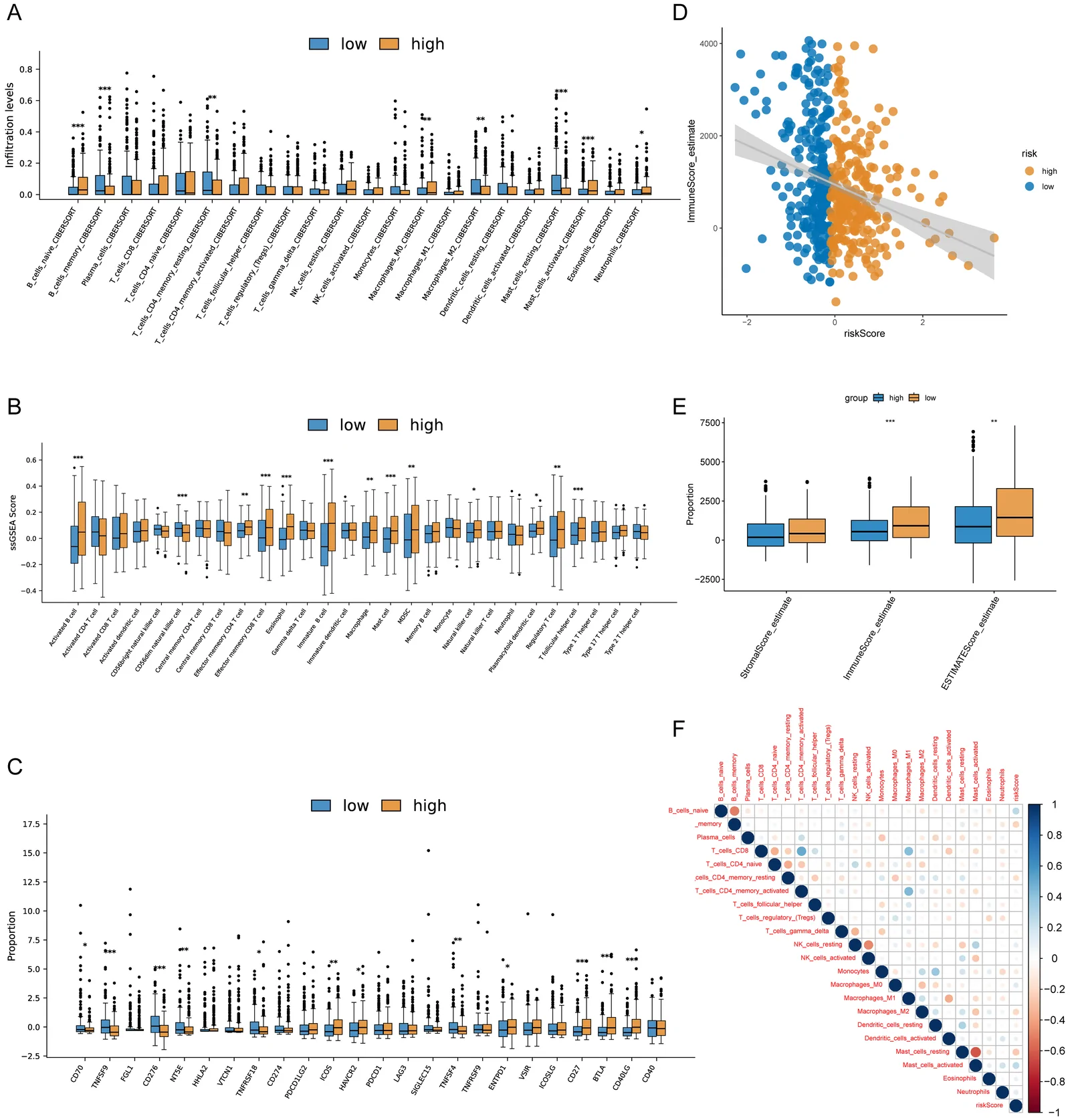
Immune infiltration landscape associated with the efferocytosis-related risk score in LUAD. **(A–C)** Boxplots showing the relative abundance of immune cell populations estimated by ssGSEA and CIBERSORT across low- and high-risk groups. Low-risk tumors exhibited higher levels of activated CD8^+^ T cells, activated CD4^+^ memory T cells, B cells, NK cells, and dendritic cells. **(D)** Correlation between immune score and risk score (Spearman correlation; gray band denotes 95% CI). **(E)** ssGSEA enrichment of representative immune-related pathways (e.g., antigen-presenting cell co-stimulation, IFN-γ response, HLA expression) in each risk group. **(F)** Correlation heatmap depicting relationships among immune cell subsets; positive correlations in blue, negative in red. * indicates p < 0.05, ** indicates p < 0.01, and *** indicates p < 0.001.

To further dissect the immune landscape underlying prognostic divergence, we compared immune cell infiltration between risk groups using multiple computational frameworks. In the low-risk subgroup, ssGSEA and CIBERSORT analyses consistently revealed greater infiltration of CD8^+^ T cells, activated CD4^+^ memory T cells, B cells, dendritic cells, and natural killer (NK) cells (**Figures 5A–C**). These immune subsets constitute key effectors of antitumor immunity and are central to cytotoxic and antigen-presenting cascades. Elevated immune and stromal scores (**Figures 5D, E**) further indicated an inflamed tumor microenvironment (“hot” phenotype) characterized by dense immune cell infiltration and heightened immune reactivity. Notably, correlation analysis demonstrated a significant negative association between the risk score and immune score (R = –0.47, *p* < 0.001; **Figure 5D**), suggesting that diminished immune infiltration parallels poorer clinical outcomes.

Conversely, the high-risk group exhibited relative depletion of effector lymphocytes and an immune milieu enriched in myeloid-derived suppressor cells (MDSCs), macrophages, and regulatory T cells (Tregs)—cell populations known to attenuate cytotoxic T-cell activity and facilitate immune evasion. Cross-correlation analysis of immune cell proportions (**Figure 5F**) revealed that T- and B-cell populations were strongly co-regulated in the low-risk group but largely decoupled in the high-risk group, indicating impaired coordination of adaptive immune responses.

At the gene level, several model genes—including *OSCAR, CD68, KLF4, SH3BP5*, and *IER5L*—displayed strong positive correlations with immune infiltrates such as activated dendritic cells, effector memory CD8^+^ T cells, and NK cells (**Supplementary Figure 5A**). This relationship reinforces their function as molecular surrogates of immune activation within the efferocytosis network. Collectively, the immune profiling results delineate a dichotomy wherein low-risk tumors exhibit an immune-inflamed, antigen-presenting phenotype conducive to effective antitumor immunity, whereas high-risk tumors display an immune-deserted or immunosuppressive microenvironment—providing a mechanistic explanation for the pronounced survival disparity between the two groups.

### Higher efferocytosis-related risk predicts inferior immunotherapy benefit and points to druggable vulnerabilities

**Figure 6:**
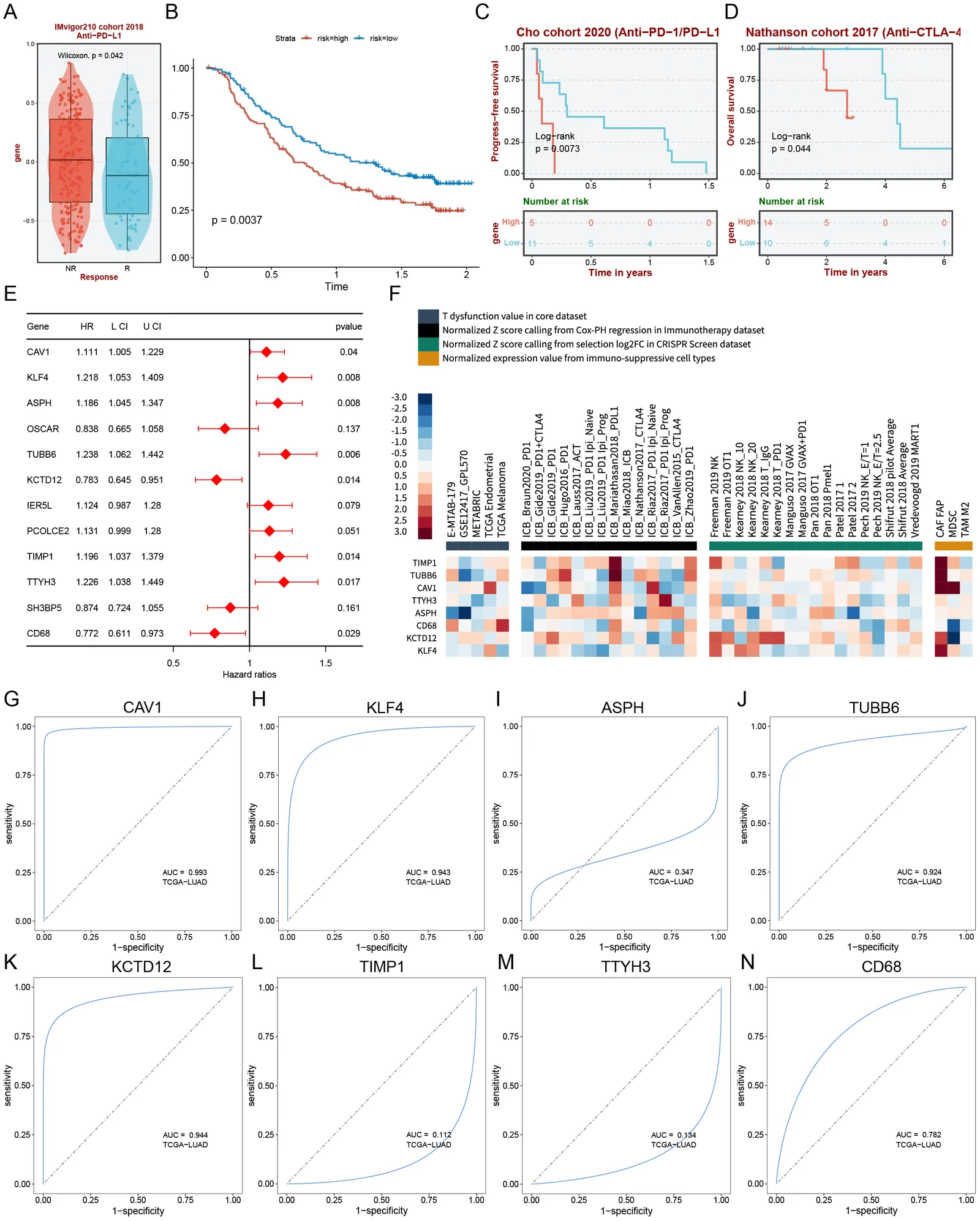
The efferocytosis-related risk score predicts immunotherapy outcomes and maps gene-level correlates. **(A)** IMvigor210 (anti-PD-L1): violin/box plot comparing risk scores between non-responders (NR) and responders (R) (Wilcoxon *p* = 0.042). **(B)** IMvigor210: Kaplan–Meier curves showing poorer survival of the high-risk group during immunotherapy (log-rank *p* = 0.0037). **(C, D)** External immunotherapy cohorts (Cho 2020, anti-PD-1/PD-L1; Nathanson 2017, anti-CTLA-4) demonstrating inferior outcomes for high-risk patients (log-rank *p* as indicated). **(E)** Univariable Cox forest plot for model genes in TCGA-LUAD identifying significant hazardous (e.g., KLF4, ASPH, TUBB6, SH3BP5, CAV1) and protective (e.g., CD68, KCTD12) factors. **(F)** TIDE-based meta-analysis heatmaps: correlations of significant genes **(E)** with T-cell dysfunction/immune-checkpoint modules across immunotherapy datasets and immunosuppressive cell signatures. **(G–N)** ROC curves of significant genes in TCGA-LUAD indicating diagnostic performance (examples: KCTD12 AUC ≈ 0.94; CAV1 AUC ≈ 0.89; TUBB6 AUC ≈ 0.82; CD68 AUC ≈ 0.78).

We next evaluated whether the efferocytosis-derived risk score predicts response to immune checkpoint blockade. In the IMvigor210 cohort (anti–PD-L1), non-responders exhibited significantly higher risk scores than responders (Wilcoxon *p* = 0.042; **Figure 6A**), and the high-risk group experienced markedly worse overall survival under immunotherapy (log-rank *p* = 0.0037; **Figure 6B**). Consistent patterns were observed in two independent datasets—Cho 2020 (anti–PD-1/PD-L1; log-rank *p* = 0.0073) and Nathanson 2017 (anti–CTLA-4)—in which high-risk patients likewise demonstrated inferior outcomes (**Figures 6C, D**). These findings indicate that elevated efferocytosis-related risk is associated with diminished clinical benefit from immune checkpoint blockade.

To identify potential molecular contributors, we performed univariate Cox regression analyses for the signature genes in TCGA-LUAD. Several genes were individually prognostic (**Figure 6E**), including *KLF4, ASPH, TUBB6, SH3BP5*, and *CAV1* (hazardous; HR > 1, *p* < 0.05), as well as *CD68* and *KCTD12* (protective; HR < 1, *p* < 0.05). Using TIDE and independent immunotherapy datasets, these significant genes exhibited consistent associations with immune-checkpoint and T-cell–dysfunction modules (**Figure 6F**), suggesting that the risk program reflects an exhausted or attenuated antitumor immune state. Diagnostic ROC analyses in TCGA-LUAD further demonstrated strong discriminative performance for several genes (e.g., *KCTD12* AUC ≈ 0.94; *CAV1* AUC ≈ 0.89; *TUBB6* AUC ≈ 0.82; *CD68* AUC ≈ 0.78), whereas others showed moderate accuracy (**Figures 6G–N**).

Finally, we explored therapeutic avenues aligned with the risk-associated biology. Across LUAD datasets, correlation profiling identified potential sensitivities of the risk program to MEK inhibitors (selumetinib, refametinib, CI-1040), PI3K/AKT/mTOR pathway inhibitors (pictilisib, dactolisib, temsirolimus), DNA-damage/DNA-PK–axis agents (NU7441), and JAK pathway inhibitors, while relative resistance patterns emerged for multiple HDAC inhibitors (e.g., panobinostat, trichostatin A) and EGFR/ERBB blockade (afatinib) (**Supplementary Figure 6A**). Collectively, these results suggest that high-risk patients—who exhibited poorer survival and less favorable outcomes in the analyzed immunotherapy-treated datasets—may possess therapeutically relevant vulnerabilities within the MAPK and PI3K–mTOR pathways, although these associations require experimental and clinical validation.

### KCTD12 expression is associated with clinical outcomes in immunotherapy-treated cohorts

**Figure 7:**
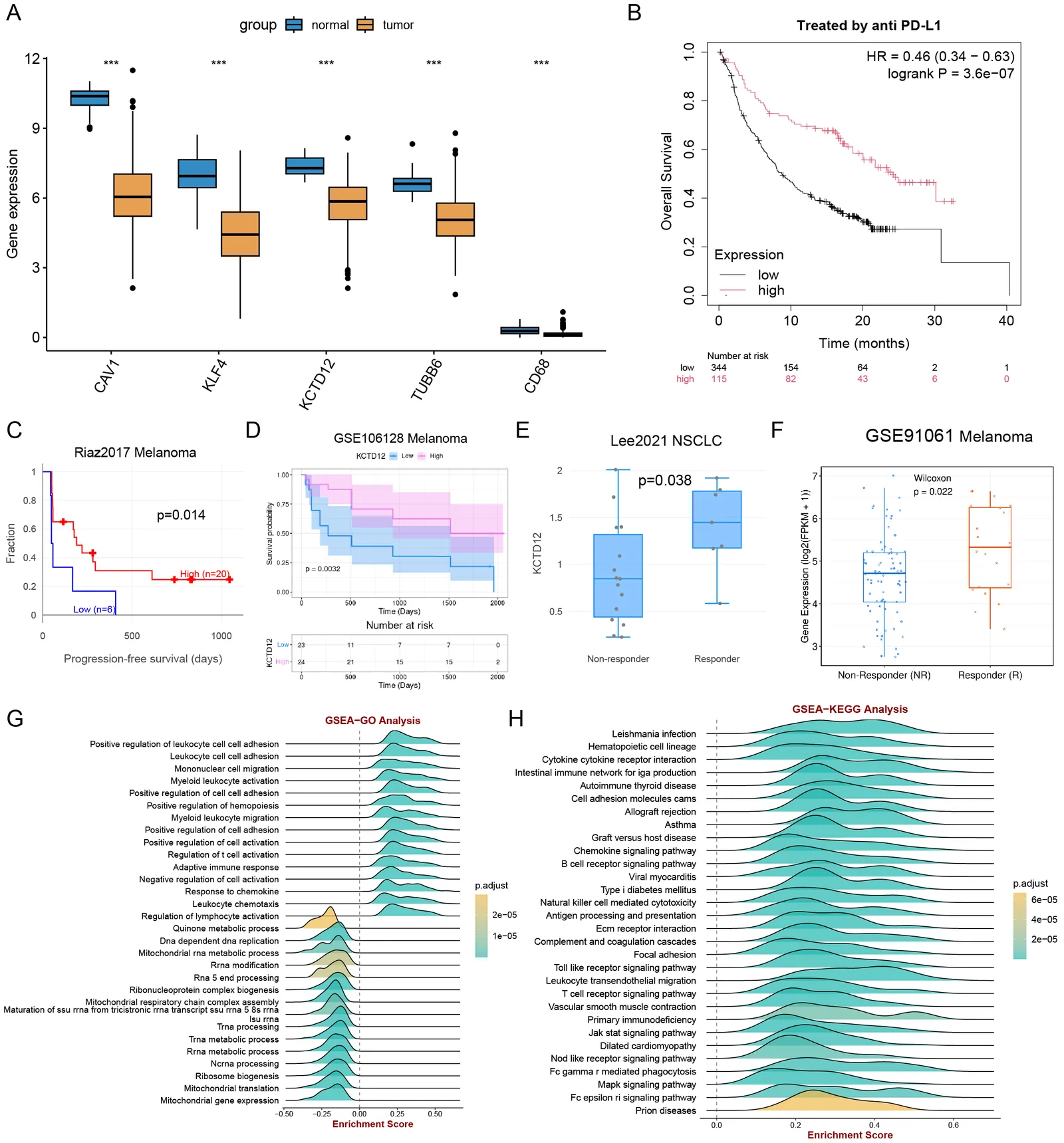
Association of KCTD12 expression with an immune-active phenotype and clinical outcomes in immunotherapy-treated cohorts. **(A)** Expression of five model genes (CAV1, KLF4, KCTD12, TUBB6, CD68) in tumor vs. normal tissues from TCGA-LUAD. **(B)** Kaplan–Meier analysis of overall survival in anti–PD-L1–treated patients (stratified by KCTD12 median expression). **(C, D)** Validation in independent immunotherapy cohorts (Riaz2017 and GSE106128, melanoma) showing longer progression-free survival in high-KCTD12 patients. **(E, F)** Boxplots comparing KCTD12 expression between responders and non-responders in Lee2021 NSCLC and GSE91061 melanoma cohorts. **(G, H)** GSEA of GO and KEGG pathways enriched in high-KCTD12 tumors, emphasizing immune activation (e.g., *T-cell receptor signaling*, *antigen presentation*, *chemokine-mediated signaling*, *B-cell receptor signaling*). *** indicates p < 0.001.

To identify key genes driving the efferocytosis-related risk model, we focused on five candidates exhibiting robust diagnostic performance (AUC > 0.70 in **Figure 6**), namely *CAV1, KLF4, KCTD12, TUBB6*, and *CD68*. In the TCGA-LUAD cohort, all five genes displayed significantly lower expression in tumor tissues compared with adjacent normal counterparts (**Figure 7A**). Given its strong protective prognostic association (HR < 1; **Figure 6E**), excellent classification accuracy (AUC = 0.94; **Figure 6K**), and clear expression gradient, *KCTD12* was selected for further investigation.

We next examined the association between KCTD12 expression and clinical outcomes across several immunotherapy-treated cohorts that differed in tumor type, therapeutic regimen, sequencing platform, and outcome definition. In the combined anti–PD-L1-treated cohort, patients with elevated KCTD12 expression exhibited prolonged overall survival (HR = 0.46, 95% CI: 0.34–0.63; log-rank p = 3.6 × 10^-7^; **Figure 7B**). Associations in the same direction were observed in the Riaz2017 melanoma cohort and the GSE106128 melanoma cohort, in which higher KCTD12 expression was associated with longer progression-free survival (p = 0.014 and p = 0.032, respectively; **Figures 7C, D**). In the Lee2021 NSCLC and GSE91061 melanoma datasets, responders exhibited higher KCTD12 expression than non-responders (p = 0.038 and p = 0.022, respectively; **Figures 7E, F**). These findings indicate that KCTD12 expression is associated with response status and survival outcomes within the analyzed immunotherapy-treated cohorts. However, because these datasets lacked matched non-immunotherapy control groups and differed substantially in clinical and technical characteristics, the analyses do not establish KCTD12 as a treatment-specific predictive biomarker.

Functional enrichment analysis further substantiated this interpretation. GO and KEGG analyses of tumors with high *KCTD12* expression revealed enrichment of immune activation and antigen presentation processes, including positive regulation of leukocyte activation, lymphocyte differentiation, antigen processing and presentation, T-cell receptor signaling, and chemokine signaling pathways (**Figures 7G, H**). Notably, mitochondrial and metabolic pathways were also upregulated, consistent with enhanced metabolic fitness of immune effector cells. Collectively, these results indicate that higher KCTD12 expression is associated with an immune-active phenotype and more favorable outcomes in the analyzed checkpoint blockade-treated cohorts. KCTD12 should therefore be considered a candidate immune-associated biomarker rather than a validated treatment-specific predictor, particularly given the heterogeneity of the included tumor types and therapeutic regimens.

### Single-cell analysis reveals that KCTD12 is associated with an immune-active and less suppressive tumor microenvironment

**Figure 8:**
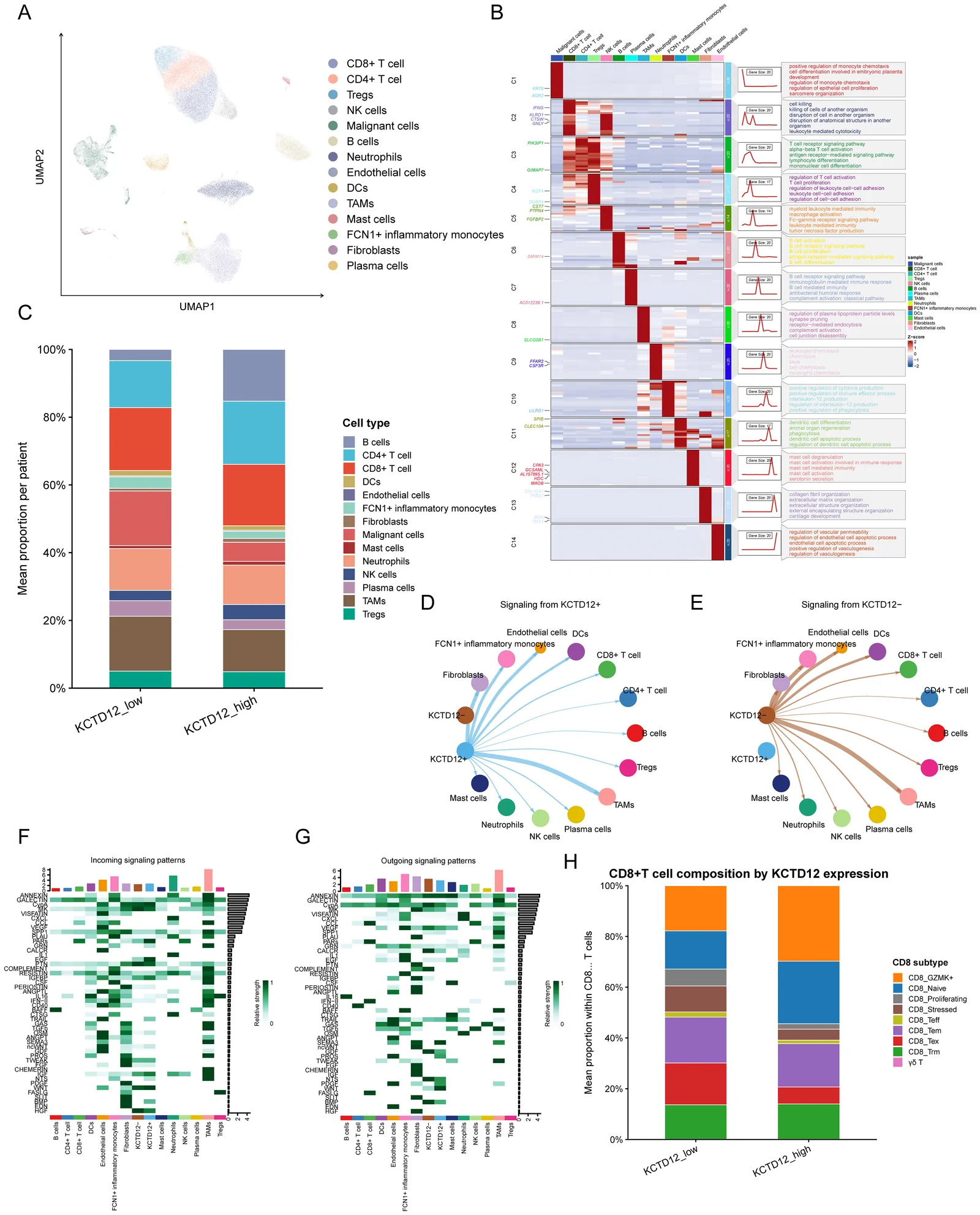
Single-cell characterization of KCTD12-associated cellular and intercellular communication features in an NSCLC immunotherapy cohort. **(A)** UMAP visualization of the major annotated cell populations, including CD8^+^ T cells, CD4^+^ T cells, regulatory T cells, NK cells, malignant cells, B cells, neutrophils, endothelial cells, dendritic cells, tumor-associated macrophages, mast cells, FCN1^+^ inflammatory monocytes, fibroblasts, and plasma cells. Each point represents one cell, and colors indicate annotated cell identity. **(B)** Heatmap showing representative marker-gene expression across the annotated cell populations. Rows represent selected marker genes or gene modules, and columns represent cell types. The accompanying enrichment annotations summarize representative biological processes associated with each transcriptional cluster. Expression values were scaled by gene before visualization. **(C)** Mean proportional composition of the major cell populations in the KCTD12-low and KCTD12-high sample groups. Proportions were calculated at the sample level before group summarization rather than by treating individual cells as independent biological replicates. **(D, E)** CellChat-inferred communication networks from KCTD12-positive malignant cells **(D)** and KCTD12-negative malignant cells **(E)** to immune and stromal cell populations. Nodes represent cell populations; edge width indicates the inferred communication strength between cell types. These networks represent computationally inferred ligand–receptor interactions and do not establish direct functional signaling. **(F)** Relative incoming signaling patterns across the annotated cell populations. Rows indicate signaling pathways and columns indicate recipient cell populations; darker color represents greater relative incoming signaling strength. **(G)** Relative outgoing signaling patterns across the annotated cell populations. Rows indicate signaling pathways and columns indicate sender cell populations; darker color represents greater relative outgoing signaling strength. **(H)** Mean composition of CD8^+^ T-cell subtypes in KCTD12-low and KCTD12-high samples, including naïve, proliferating, stressed, effector-memory, central-memory, γδ T-cell, and GZMK^+^ subsets. The stacked bars summarize sample-level subtype proportions. These analyses characterize associations between KCTD12 expression and the NSCLC immune microenvironment but do not constitute LUAD-specific validation. Single-cell analysis associates malignant-cell KCTD12 expression with an immune-active and relatively less suppressive cellular context in NSCLC. **(A)** Uniform manifold approximation and projection (UMAP) of cells from the NSCLC immunotherapy single-cell RNA-sequencing dataset GSE207422, colored according to the annotated major cell populations. Cell identities were assigned according to the original dataset annotations and supported by canonical lineage-marker expression. The annotated populations included CD8^+^ T cells, CD4^+^ T cells, regulatory T cells (Tregs), natural killer (NK) cells, malignant cells, B cells, neutrophils, endothelial cells, dendritic cells (DCs), tumor-associated macrophages (TAMs), mast cells, FCN1^+^ inflammatory monocytes, fibroblasts, and plasma cells. **(B)** Heatmap showing row-scaled average expression of representative marker genes across the annotated cell populations. Selected Gene Ontology terms associated with the cluster-specific transcriptional programs are shown on the right. Marker expression was used to support cell-type annotation rather than as the sole criterion for identifying malignant cells. **(C)** Mean cellular composition of tumors stratified according to patient-level malignant-cell KCTD12 expression. Raw counts from malignant cells were aggregated within each patient to generate pseudobulk profiles, and patients were divided at the median pseudobulk KCTD12 expression into KCTD12-low (n = 7) and KCTD12-high (n = 8) groups. Bars show the group-level mean proportions of the indicated cell populations and are presented as a descriptive summary; individual cells were not treated as independent biological replicates. **(D, E)** CellChat-inferred communication networks depicting outgoing signaling from KCTD12-positive **(D)** and KCTD12-negative **(E)** malignant-cell populations to immune and stromal cell populations. Edge width represents the relative inferred communication strength between the indicated cell populations. These networks represent computationally inferred ligand–receptor communication rather than experimentally validated intercellular interactions. **(F, G)** Heatmaps summarizing the relative incoming **(F)** and outgoing **(G)** signaling patterns across malignant, immune, and stromal cell populations. Rows represent signaling pathways, columns represent cell populations, and color intensity indicates the relative pathway-specific signaling strength inferred by CellChat. **(H)** Mean composition of transcriptionally defined CD8^+^ T-cell subtypes in the KCTD12-low and KCTD12-high patient groups. Subtypes included GZMK^+^, naïve, proliferating, stressed, effector, effector-memory, exhausted, tissue-resident memory, and γδ T-cell states. The stacked bars provide a descriptive group-level summary of the relative abundance of each subtype within the CD8^+^ T-cell compartment. Patient-level comparisons were performed using two-sided Wilcoxon rank-sum tests with Benjamini–Hochberg correction and are reported in **Supplementary Figure S5**; no individual cell was treated as an independent replicate.

To characterize the cellular context associated with KCTD12 expression during lung cancer immunotherapy, we analyzed the GSE207422 single-cell RNA-sequencing dataset from patients with NSCLC treated with neoadjuvant immunotherapy. Following quality control, normalization, dimensionality reduction, and cell-type annotation, we identified the major malignant, immune, and stromal populations, including malignant cells, CD8^+^ T cells, CD4^+^ T cells, regulatory T cells, NK cells, B cells, plasma cells, neutrophils, FCN1^+^ inflammatory monocytes, tumor-associated macrophages, dendritic cells, mast cells, endothelial cells, and fibroblasts (**Figure 8A**). The assigned cellular identities were supported by canonical lineage-marker expression (**Supplementary Figure 7A**), while marker-gene and functional enrichment analyses revealed transcriptional programs related to lymphocyte activation, antigen presentation, myeloid inflammation, epithelial biology, and stromal remodeling (**Figure 8B**). Malignant cells were defined according to the original GSE207422 annotation and were further supported by the expression of epithelial and tumor-associated markers, including KRT8, KRT18, and KRT19.

The cellular-composition analysis was conducted using patients as the biological replicates. To define patient-level KCTD12 expression, raw counts from malignant cells belonging to the same patient were aggregated into pseudobulk profiles. Patients represented by fewer than 20 malignant cells were excluded, leaving 15 independent patients for analysis. Patients were divided at the median malignant-cell KCTD12 expression into KCTD12-low (n = 7) and KCTD12-high (n = 8) groups (**Supplementary Figure 7B**). Cell-type proportions were calculated independently for each patient, rather than by treating individual cells as independent observations.

At the patient level, the KCTD12-high group exhibited a coordinated compositional pattern characterized by higher median proportions of several lymphocyte populations, including B cells, NK cells, CD4^+^ T cells, and CD8^+^ T cells, together with lower median proportions of FCN1^+^ inflammatory monocytes, fibroblasts, tumor-associated macrophages, and malignant cells (**Supplementary Figure 7C**). Although no individual cell-type comparison remained significant after Benjamini–Hochberg correction, the direction of these changes was broadly consistent across multiple immune, myeloid, stromal, and malignant compartments. Continuous correlation analysis provided a concordant pattern: patient-level malignant-cell KCTD12 expression was positively associated with the proportions of B cells, NK cells, and CD4^+^ and CD8^+^ T cells, whereas inverse associations were observed with FCN1^+^ inflammatory monocytes, fibroblasts, tumor-associated macrophages, and malignant cells (**Supplementary Figure 7D**). These correlations did not individually remain significant after multiple-testing correction, reflecting the limited number of independent patients, but their coordinated directionality was consistent with an immune-enriched and relatively less myeloid–stromal-dominant tumor microenvironment.

We next explored whether the cellular context associated with KCTD12 was accompanied by differences in predicted tumor–immune communication. CellChat analysis suggested that KCTD12-positive malignant cells exhibited stronger predicted interactions with dendritic cells, CD8^+^ T cells, and CD4^+^ T cells, whereas KCTD12-negative malignant cells showed stronger predicted interactions with tumor-associated macrophages, neutrophils, regulatory T cells, and fibroblasts (**Figures 8D, E**). Incoming and outgoing signaling analyses further indicated distinct ligand–receptor communication programs associated with KCTD12-positive and KCTD12-negative malignant cells (**Figures 8F, G**). These results were interpreted as computationally inferred communication patterns rather than direct evidence that KCTD12 causally regulates intercellular signaling. Nevertheless, the predicted communication networks were directionally concordant with the patient-level cellular-composition analysis.

Because the functional state of CD8^+^ T cells is closely related to immunotherapy outcome, we further reclustered the CD8^+^ T-cell compartment and annotated GZMK^+^, naïve, proliferating, stressed, effector, effector-memory, exhausted, tissue-resident memory, and γδ T-cell populations based on canonical marker expression (**Supplementary Figure 7E**). The KCTD12-high group showed higher median proportions of GZMK^+^, naïve, and tissue-resident memory CD8^+^ T-cell states and lower median proportions of exhausted, stressed, and proliferating CD8^+^ T-cell states (**Figure 8H**; **Supplementary Figure 7F**). Although these individual subtype comparisons did not remain significant after multiple-testing correction, the overall directional pattern was consistent with a CD8^+^ T-cell compartment displaying relatively fewer dysfunctional features.

Collectively, the patient-level compositional analysis, continuous correlation analysis, CD8^+^ T-cell-state profiling, and predicted cell–cell communication patterns.

### KCTD12 suppresses tumor cell proliferation and correlates with cytotoxic T-cell activation

**Figure 9:**
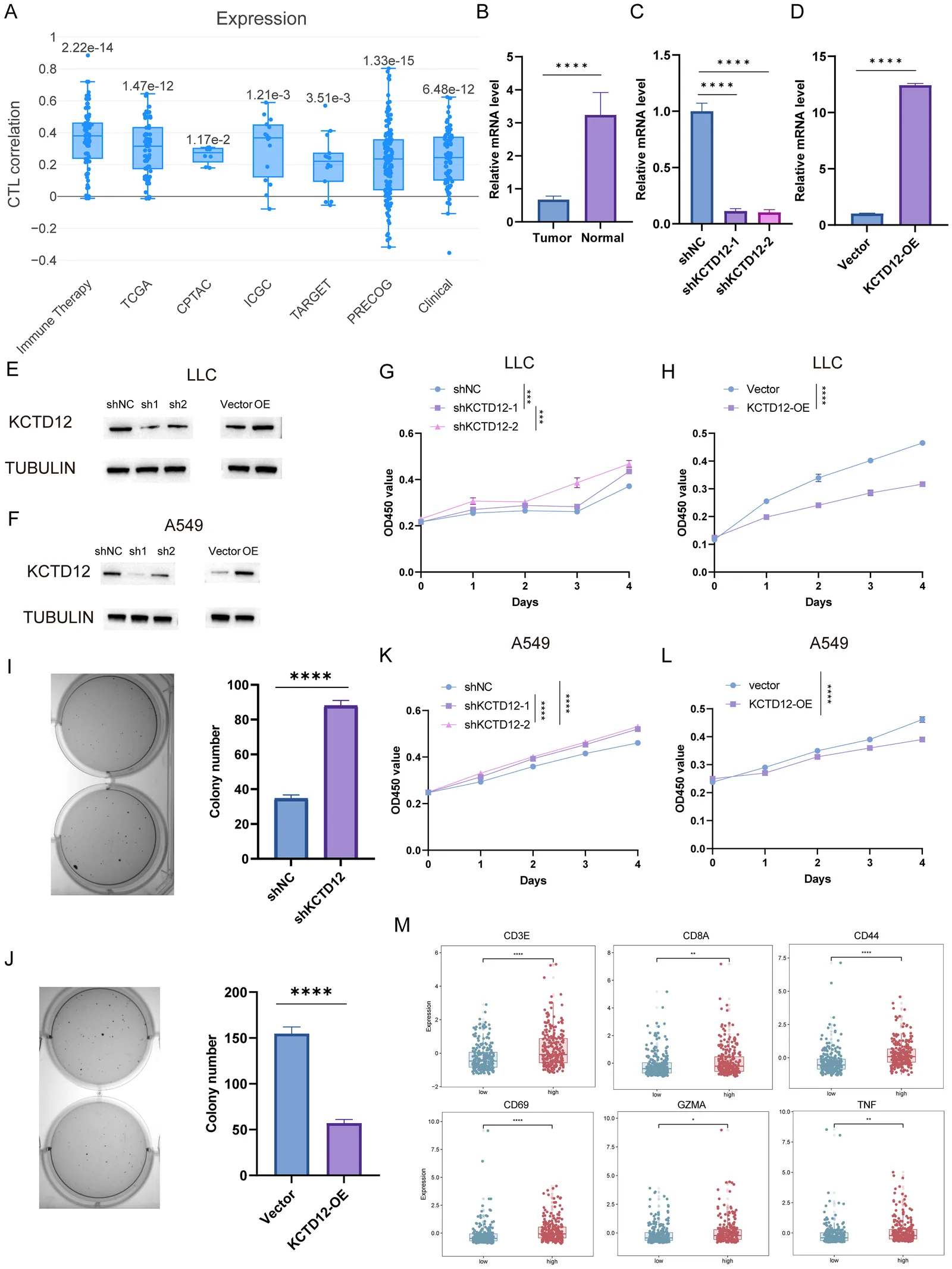
KCTD12 suppresses tumor cell proliferation and correlates with cytotoxic T-cell activation in LUAD. **(A)** Correlation of KCTD12 expression with cytotoxic T lymphocyte (CTL) signatures across six public cohorts (CIDE database). **(B)** qRT–PCR analysis of KCTD12 mRNA expression in paired LUAD tumor and adjacent normal tissues (n = 30 pairs). **(C, D)** Verification of KCTD12 knockdown (shKCTD12-1, shKCTD12-2) and overexpression (KCTD12-OE) efficiency in LLC cells. **(E, F)** Representative Western blots and densitometric quantification confirming KCTD12 protein depletion and overexpression in LLC/A549 cells. **(G)** CCK-8 proliferation assay showing that KCTD12 knockdown enhanced LLC cell proliferation compared with shNC control cells. **(H)** CCK-8 proliferation assay showing that KCTD12 overexpression suppressed LLC cell proliferation compared with vector control cells. **(I)** Colony formation assay showing that KCTD12 knockdown increased colony-forming ability of LLC cells. Representative colony images and quantitative analysis are shown. **(K)** Colony formation assay showing that KCTD12 overexpression reduced colony-forming ability of LLC cells. Representative colony images and quantitative analysis are shown. **(K)** CCK-8 proliferation assay showing that KCTD12 knockdown enhanced A549 cell proliferation compared with shNC control cells. **(L)** CCK-8 proliferation assay showing that KCTD12 overexpression suppressed A549 cell proliferation compared with vector control cells. **(M)** Expression of CD8^+^ T-cell activation and cytotoxicity-related markers, including CD3E, CD8A, CD44, CD69, GZMA, and TNF, in TCGA-LUAD samples stratified by KCTD12 expression level. High KCTD12 expression was associated with increased expression of cytotoxic T-cell effector markers. Data are presented as mean ± SD where applicable. Statistical significance was determined using two-tailed Student’s t-test or one-way ANOVA. *p < 0.05; **p < 0.01; ***p < 0.001; ****p < 0.0001.

To validate the functional relevance of KCTD12, we first examined its relationship with cytotoxic T lymphocyte activity using the CIDE database. KCTD12 expression was positively correlated with CTL signatures across multiple independent datasets, including immunotherapy cohorts, TCGA, CPTAC, ICGC, TARGET, PRECOG, and clinical datasets (**Figure 9A**). This consistent association supports the potential role of KCTD12 as a marker of cytotoxic immune activation.

We then assessed KCTD12 expression in clinical LUAD specimens. qRT-PCR analysis of paired LUAD tumor and adjacent normal tissues showed that KCTD12 was significantly downregulated in tumor tissues compared with normal tissues (**Figure 9B**). This expression pattern is consistent with its protective prognostic association and suggests a potential tumor-suppressive role in LUAD.

To experimentally investigate the biological function of KCTD12, we established KCTD12 knockdown and overexpression models in LLC cells. qRT-PCR and western blot confirmed efficient knockdown of KCTD12 by two independent shRNAs and robust overexpression in KCTD12-OE cells (**Figures 9C–E**). Functionally, KCTD12 knockdown significantly promoted LLC cell proliferation, whereas KCTD12 overexpression suppressed cell growth, as shown by CCK-8 assays (**Figures 9G, H**). Colony formation assays further confirmed this phenotype: KCTD12 silencing markedly increased colony-forming ability, whereas KCTD12 overexpression significantly reduced colony formation (**Figures 9I, J**). To determine whether these findings were reproducible in a human LUAD model, we performed analogous experiments in A549 cells. Genetic perturbation of KCTD12 was confirmed at protein levels (**Figure 9F**). Consistent with the LLC results, KCTD12 knockdown enhanced A549-cell proliferation, whereas KCTD12 overexpression suppressed the phenotype (**Figures 9K, L**). These results demonstrate that KCTD12 restrains the proliferative capacity of lung cancer cells.

Consistent with the immune-activating role suggested by our single-cell analysis, TCGA-LUAD tumors with high KCTD12 expression exhibited significantly elevated expression of CD8^+^ T-cell activation and cytotoxicity-related markers, including CD3E, CD8A, CD44, CD69, GZMA, and TNF (**Figure 9M**). Together, these findings indicate that KCTD12 not only suppresses tumor cell proliferation but is also associated with a cytotoxic T-cell–inflamed tumor microenvironment.

### Spatial transcriptomics and co-culture assays confirm that KCTD12 expression is associated with immunotherapy response and enhances CD8^+^ T-cell cytotoxicity

**Figure 10:**
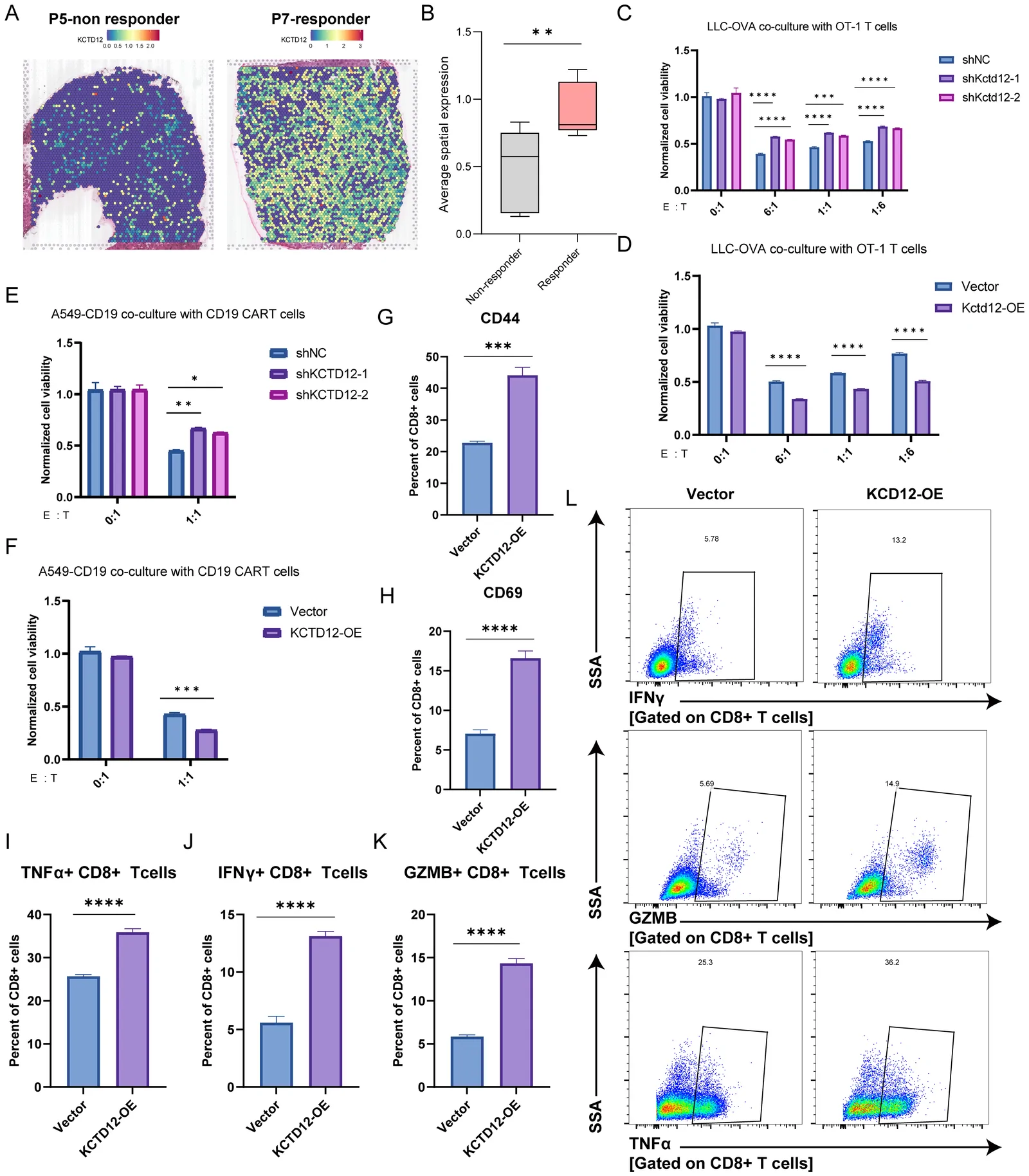
KCTD12 expression is associated with immunotherapy response and modulates OT-1 CD8^+^ T-cell-mediated tumor killing. **(A)** Spatial transcriptomic visualization of KCTD12 expression in representative non-responder and responder samples from an immunotherapy-treated cohort. **(B)** Quantification of average spatial KCTD12 expression for each sample between non-responders and responders, showing significantly elevated KCTD12 expression in responders. **(C)** Cell viability assay of LLC-OVA cells co-cultured with OT-1 CD8^+^ T cells at different effector-to-target ratios after KCTD12 knockdown. **(D)** Cell viability assay of LLC-OVA cells co-cultured with OT-1 CD8^+^ T cells at different effector-to-target ratios after KCTD12 overexpression. **(E)** Cell viability assay of A549-CD19 cells co-cultured with CD19-CAR T cells at different effector-to-target ratios after KCTD12 knockdown. **(F)** Cell viability assay of A549-CD19 cells co-cultured with CD19-CAR T cells at different effector-to-target ratios after KCTD12 overexpression. **(G, H)** Flow cytometric quantification of CD44^+^ and CD69^+^ CD8^+^ T cells after co-culture with vector- or KCTD12-overexpressing LLC-OVA cells. **(I–K)** Flow cytometric quantification of TNFα^+^, IFNγ^+^, and GZMB^+^ CD8^+^ T cells after co-culture. KCTD12 overexpression significantly increased cytokine-producing and granzyme B-positive CD8^+^ T-cell populations, indicating enhanced cytotoxic effector function. **(L)** Representative flow cytometry plots showing increased IFNγ^+^, GZMB^+^, and TNFα^+^ CD8^+^ T cells in the KCTD12-overexpression group compared with the vector control group. Cells were gated on CD8^+^ T cells. Data are presented as mean ± SD. Statistical significance was determined using two-tailed Student’s t-test or one-way ANOVA. **p < 0.01; ***p < 0.001; ****p < 0.0001.

To further determine whether KCTD12 is associated with immunotherapy responsiveness *in situ*, we analyzed spatial transcriptomic data from immunotherapy-treated HCC tumor samples. Spatial visualization showed higher KCTD12 expression in the responder sample compared with the non-responder sample (**Figure 10A**). Quantitative analysis of average spatial expression confirmed that KCTD12 was significantly enriched in responders (**Figure 10B**), supporting that KCTD12 exhibits spatial associations with the immune microenvironment in the context of immune checkpoint blockade.

We next investigated whether tumor-cell KCTD12 expression influenced susceptibility to antigen-specific T-cell killing. LLC-OVA cells with KCTD12 knockdown or overexpression were co-cultured with activated OT-1 CD8^+^ T cells at effector-to-target ratios of 6:1, 1:1, and 1:6. For each KCTD12 condition, residual tumor-cell survival after co-culture was normalized to the corresponding tumor-cell-only control to account for differences in baseline proliferation. KCTD12 depletion increased the residual viable fraction of LLC-OVA cells following OT-1 exposure, whereas KCTD12 overexpression reduced residual tumor-cell survival across the tested E:T ratios (**Figures 10C, D**), suggesting tumor-cell KCTD12 expression was associated with enhanced susceptibility to CD8+ T-cell-mediated killing and with increased CD8+ T-cell effector features in the co-culture system. To determine whether this association extended to a human tumor-cell and T-cell model, we established A549 cells expressing CD19 and co-cultured them with human CD19-directed CAR-T cells. KCTD12 overexpression reduced, whereas KCTD12 depletion increased, the residual viable A549-CD19 tumor-cell fraction after CAR-T-cell exposure (**Figures 10E, F**).

Flow cytometric analysis further demonstrated that KCTD12 overexpression promoted CD8^+^ T-cell activation and effector function during co-culture. Compared with vector control cells, KCTD12-overexpressing LLC-OVA cells induced significantly higher proportions of CD44^+^ and CD69^+^ CD8^+^ T cells (**Figures 10G, H**). In addition, the percentages of TNFα^+^, IFNγ^+^, and GZMB^+^ CD8^+^ T cells were markedly increased in the KCTD12-overexpression group (**Figures 10I–K**). Representative flow cytometry plots confirmed the enhanced production of IFNγ, GZMB, and TNFα in CD8^+^ T cells co-cultured with KCTD12-overexpressing tumor cells (**Figure 10L**).

Collectively, the spatial data support an association between KCTD12 expression and response status in immunotherapy-treated samples, whereas the *in vitro* experiments demonstrate that KCTD12 modulation affects tumor-cell proliferation, susceptibility to antigen-specific CD8^+^ T-cell killing, and CD8^+^ T-cell activation. These complementary findings do not, however, establish KCTD12 as a treatment-specific predictive biomarker.

## Discussion

Our comprehensive analysis delineates a previously uncharacterized efferocytosis-related transcriptional landscape in LUAD and underscores its profound implications for tumor immunity and patient prognosis. By integrating large-scale transcriptomic datasets, we identified two molecular subtypes and established a robust efferocytosis-derived prognostic signature that remained predictive across multiple independent cohorts. Patients classified as high-risk exhibited inferior survival and less favorable outcomes in the analyzed immunotherapy-treated datasets, together with a tumor microenvironment characterized by immune suppression and metabolic reprogramming. In contrast, low-risk tumors displayed an immune-inflamed phenotype. Because the immunotherapy analyses were conducted in heterogeneous treated cohorts without matched untreated comparators, these findings should be interpreted as outcome associations rather than evidence of treatment-specific predictive utility.

Mechanistically, the low-risk subgroup was enriched in antigen presentation, interferon signaling, and immune activation pathways, indicating preserved immunogenicity and efficient T-cell recruitment. In contrast, the high-risk subgroup was characterized by proliferative and pro-metastatic hallmarks—such as E2F, MYC, EMT, and mTORC1 signaling—along with recurrent *TP53* and *KRAS* mutations, genomic alterations classically associated with immune exclusion and diminished efficacy of checkpoint blockade. These findings suggest that dysregulated efferocytosis may orchestrate a shift from immune surveillance to immune tolerance, thereby facilitating immune evasion and therapy resistance.

At the gene level, KCTD12 emerged as a pivotal determinant linking the efferocytosis-related risk program to antitumor immunity. Unlike genes that simply reflect tumor-intrinsic aggressiveness, KCTD12 appeared to mark an immune-reactive and less suppressive tumor ecosystem. Single-cell analysis of an NSCLC immunotherapy cohort showed that KCTD12-high tumors contained a higher proportion of immune cells and fewer malignant cells and TAMs. More importantly, KCTD12-positive malignant cells preferentially communicated with dendritic cells and CD8^+^/CD4^+^ T cells, whereas KCTD12-negative malignant cells displayed stronger interactions with TAMs, neutrophils, Tregs, and fibroblasts. These findings suggest that KCTD12 expression may shift tumor-cell communication away from suppressive myeloid–stromal circuits and toward adaptive immune engagement. The CD8^+^ T-cell compartment further supported this interpretation. KCTD12-high tumors contained more GZMK^+^ CD8^+^ T cells and fewer stressed or exhausted CD8^+^ T-cell subsets, indicating that KCTD12 may be associated with preservation of cytotoxic T-cell functionality. This observation was consistent with bulk transcriptomic analyses showing positive correlations between KCTD12 and CTL signatures, as well as increased expression of CD8^+^ T-cell activation markers, including CD3E, CD8A, CD44, CD69, GZMA, and TNF, in KCTD12-high LUAD tumors.

Functionally, KCTD12 behaved as a tumor-suppressive and immune-potentiating factor. qRT-PCR analysis confirmed that KCTD12 was downregulated in LUAD tumor tissues compared with adjacent normal tissues. *In vitro*, KCTD12 knockdown promoted tumor cell proliferation and colony formation, whereas KCTD12 overexpression suppressed these malignant phenotypes. In antigen-specific co-culture experiments, KCTD12 silencing reduced OT-1 CD8^+^ T-cell-mediated tumor killing, while KCTD12 overexpression enhanced tumor cell susceptibility to cytotoxic T-cell attack. Flow cytometry further showed that KCTD12-overexpressing tumor cells induced higher proportions of CD44^+^, CD69^+^, TNFα^+^, IFNγ^+^, and GZMB^+^ CD8^+^ T cells. Together with spatial transcriptomic evidence showing higher KCTD12 expression in immunotherapy responders, these data support KCTD12 as a potential biomarker of immune responsiveness and tumor-cell KCTD12 expression was associated with enhanced susceptibility to CD8+ T-cell-mediated killing.

## Limitations

Several limitations should be acknowledged. First, this study is primarily based on transcriptomic and bioinformatic analyses; thus, the causal mechanisms by which efferocytosis influences immune regulation require further *in vivo* validation. Second, while functional experiments focused on KCTD12, additional efferocytosis-related genes identified by our model may also contribute to LUAD progression and merit detailed mechanistic studies. Third, the immunotherapy cohorts were heterogeneous with respect to tumor type, treatment regimen, sequencing platform, sample size, clinical characteristics, response criteria, and survival endpoint. And the GSE207422 single-cell dataset was derived from patients with NSCLC receiving neoadjuvant immunotherapy and was not restricted to LUAD. These differences may introduce substantial clinical and technical confounding. Moreover, the analyses were conducted predominantly within immunotherapy-treated populations and did not include appropriately matched non-immunotherapy control groups. We therefore could not distinguish a treatment-specific predictive effect from a general prognostic or immune-contextual association. Finally, both the LLC-OVA/OT-1 and A549-CD19/CD19-CAR-T systems rely on engineered surrogate antigens. These models permit controlled assessment of antigen-receptor-dependent T-cell cytotoxicity but do not reproduce recognition of endogenous LUAD-associated antigens. Formal establishment of predictive value would require treatment-homogeneous or randomized cohorts and a statistically significant interaction between KCTD12 status and treatment exposure. Accordingly, KCTD12 should currently be regarded as a candidate biomarker associated with outcomes in immunotherapy-treated patients rather than a validated predictor of immunotherapy benefit. Forth, direct experimental evidence demonstrating a role of KCTD12 in apoptotic-cell engulfment or other canonical efferocytosis processes is currently lacking, and future studies employing dedicated efferocytosis assays (such as fluorescent apoptotic-cell uptake assays using macrophages or professional phagocytes) will be necessary to determine whether KCTD12 directly regulates efferocytosis.

## Conclusion

In conclusion, our study identifies an efferocytosis-associated transcriptional program linked to prognosis and immune heterogeneity in LUAD. The resulting risk signature stratified patients according to survival and immune features across the analyzed cohorts. KCTD12 expression was associated with an immune-active microenvironment and more favorable outcomes in several immunotherapy-treated datasets, while functional experiments showed that KCTD12 modulates tumor-cell proliferation and CD8^+^ T-cell-mediated cytotoxicity *in vitro*. These findings support KCTD12 as a candidate immune-associated biomarker, but its treatment-specific predictive value and clinical utility require validation in prospectively designed, treatment-homogeneous, and appropriately controlled cohorts.

## Data Availability

The original contributions presented in the study are included in the article/**Supplementary Material**, further inquiries can be directed to the corresponding author/s.
