# Supplementary material for: Efferocytosis related KCTD12 is a clinico-immune target in lung adenocarcinoma
Source: Front Oncol. 2026 Aug 19;16:1930176. doi: 10.3389/fonc.2026.1930176 (PMC13533763; doi:10.3389/fonc.2026.1930176)
Supplement: Supplementary file 1 [file Supplementaryfile1.docx]

**Figure S1. Prognostic value of individual efferocytosis-related genes in TCGA-LUAD.**

(A–L) Kaplan–Meier survival curves for the 12 model genes (OSCAR, KCTD12, KLF4, CAV1, CD68, IER5L, TTC9, TUBB6, ASPH, PCOLCE2, SH3BP5, and TIMP1).

Figure S2. Multicollinearity, cutoff sensitivity, and bootstrap optimism correction in TCGA-LUAD.

(A) VIFs for the 12 signature genes; the maximum VIF was 1.689, and the maximum absolute pairwise correlation was 0.498.

(B) High- versus low-risk HRs at the 40th, 50th, and 60th percentile cutoffs.

(C) Apparent and optimism-corrected Harrell C-indexes after 1,000 bootstrap resamples.

Figure S3. External validation of the efferocytosis-related prognostic score.

(A) Cox HRs with 95% CIs comparing high- versus low-risk groups defined by each cohort’s median.

(B) Harrell C-indexes with 1,000-bootstrap 95% CIs.

(C) Cumulative/dynamic 1-, 3-, and 5-year AUCs with inverse-probability-of-censoring weighting and 1,000-bootstrap 95% CIs.

**Figure S4. Multivariate Cox regression and tumor mutation burden analysis.**

(A) Forest plot of multivariate Cox regression confirming the risk score as an independent prognostic factor (HR = 2.591, 95% CI: 2.028–3.312, *p* < 0.001).
(B) Violin plot showing comparison of tumor mutation burden (TMB) between high- and low-risk groups (Wilcoxon *p* = 0.0037).

**Figure S5. Correlation between model genes and immune-cell infiltration.**

(A) Heatmap showing correlations between the 12 efferocytosis-related model genes and 25 immune-cell subsets.

**Figure S6. Drug–risk score correlation landscape across LUAD datasets.**

(A) Heatmap summarizing correlations between the efferocytosis-related risk program and predicted drug responses. Blue denotes putative sensitivity (negative correlation of expression/risk with drug resistance) highlighting agents such as MEK inhibitors (selumetinib, refametinib, CI-1040), PI3K/mTOR inhibitors (pictilisib, dactolisib, temsirolimus), JAK3 inhibitors, and DNA-PK inhibition (NU7441). Red denotes putative resistance, enriched for HDAC inhibitors (panobinostat, trichostatin A) and ERBB inhibition (afatinib). Values are shown per dataset; color encodes correlation strength.

**Figure S7. Patient-level assessment of cell-type annotations and KCTD12-associated cellular composition in the GSE207422 NSCLC cohort.**
**(A)** Dot plot showing the expression of canonical lineage markers across the major immune, stromal, and malignant-cell populations identified in the GSE207422 single-cell RNA-sequencing dataset. Malignant cells were annotated according to the original dataset annotation and supported by the expression of epithelial and tumor-associated markers, including KRT8, KRT18, and KRT19. Dot size represents the fraction of cells expressing each gene within the indicated population, and color represents the rescaled mean expression level.
**(B)** Patient-level stratification according to KCTD12 expression in malignant cells. Raw transcript counts from malignant cells were aggregated for each patient to generate pseudobulk expression profiles, followed by normalization and logCPM transformation. Patients with fewer than 20 malignant cells were excluded. The remaining 15 patients were divided at the median patient-level KCTD12 expression into KCTD12-low (n = 7) and KCTD12-high (n = 8) groups. Each point represents one independent patient, and the dashed line indicates the median cutoff.
**(C)** Patient-level comparison of the proportions of major malignant, immune, and stromal cell populations between the KCTD12-low and KCTD12-high groups. Each point represents one independent patient. Group differences were evaluated using two-sided Wilcoxon rank-sum tests, followed by Benjamini–Hochberg correction across the tested cell populations. Adjusted p values are shown as FDR values; ns, not significant.
**(D)** Patient-level Spearman correlations between malignant-cell KCTD12 pseudobulk expression and the proportion of each major cell population. Each point represents one cell population, and the horizontal position indicates the Spearman correlation coefficient. The dashed vertical line denotes a correlation coefficient of zero. Benjamini–Hochberg correction was applied across the tested cell populations.
**(E)** Dot plot showing canonical markers used to annotate CD8⁺ T-cell states, including GZMK⁺, naïve, proliferating, stressed, effector, effector-memory, exhausted, tissue-resident memory, and γδ T-cell populations. Dot size represents the fraction of cells expressing each gene, and color represents the rescaled mean expression level.
**(F)** Patient-level comparison of CD8⁺ T-cell-subtype proportions between the KCTD12-low and KCTD12-high groups. Subtype proportions were calculated within the CD8⁺ T-cell compartment for each patient. Each point represents one independent patient. Statistical comparisons were performed using two-sided Wilcoxon rank-sum tests with Benjamini–Hochberg correction across CD8⁺ T-cell subtypes. Adjusted p values are shown as FDR values; ns, not significant; NA, adjusted p value not estimable.
